# Post-intrathecal chemotherapy-related paraplegia syndrome in hematological cancer patients: A systematic review

**DOI:** 10.1093/noajnl/vdae217

**Published:** 2024-12-20

**Authors:** Matthijs Monnikhof, Gabriella Hamming, Sandra Crnko, Rick Brandsma, Anna van Rhenen, Thomas Monnikhof, Niels Bovenschen, Gertjan Kaspers, Thijs W H Flinsenberg

**Affiliations:** Department of Pathology, University Medical Center Utrecht, Utrecht, The Netherlands; Pediatric Oncology, Princes Maxima Center, Utrecht, The Netherlands; Department of Pathology, University Medical Center Utrecht, Utrecht, The Netherlands; Pediatric Oncology, Princes Maxima Center, Utrecht, The Netherlands; Center of Haemato-Oncology, University Medical Center Utrecht, Utrecht, The Netherlands; Department of Pathology, University Medical Center Utrecht, Utrecht, The Netherlands; Center for Translational Immunology, University Medical Center Utrecht, Utrecht, The Netherlands; Department of Pathology, University Medical Center Utrecht, Utrecht, The Netherlands; Pediatric Oncology, Emma Children’s Hospital, University Medical Center, Amsterdam, The Netherlands; Pediatric Oncology, Princes Maxima Center, Utrecht, The Netherlands; Center for Translational Immunology, University Medical Center Utrecht, Utrecht, The Netherlands; Center of Haemato-Oncology, University Medical Center Utrecht, Utrecht, The Netherlands

**Keywords:** cancer, chemotherapy, hematology, intrathecal, paraplegia

## Abstract

**Background:**

Intrathecal (IT) chemotherapy is essential in treating hematological malignancies, but it can lead to ascending paraplegia, a condition that currently lacks clear management guidelines.

**Methods:**

We conducted a systematic review, analyzing 1219 studies and 116 patients, adhering to PRISMA guidelines for individual patient data. The study, registered under PROSPERO (CRD42022362121), focused on the onset, diagnostic approaches, and therapeutic interventions associated with this complication, and management strategies to tackle the ascending paraplegia.

**Results:**

Paraplegia typically manifests approximately 10 days after chemotherapy, irrespective of injection frequency. In 95% of cases, paralysis stabilizes around the umbilical region, although some patients progress to upper limb involvement and respiratory compromise. Despite various diagnostic methods, consistent inflammatory markers in blood or cerebrospinal fluid are lacking, with approximately 60% of patients showing normal magnetic resonance imaging results at presentation. Misdiagnoses often include transverse myelitis, Guillain-Barré syndrome, and autoimmune radiculitis. Common treatments such as corticosteroids and intravenous immunoglobulins show limited effectiveness.

**Conclusion:**

Our review delineates the clinical entity of ascending paraplegia following IT chemotherapy, aiming to increase clinician awareness and provide prognostic insight. We introduce the term post-IT paraplegia syndrome to facilitate accurate diagnosis and optimize treatment strategies for affected patients.

## Graphical Abstract

**Figure:**
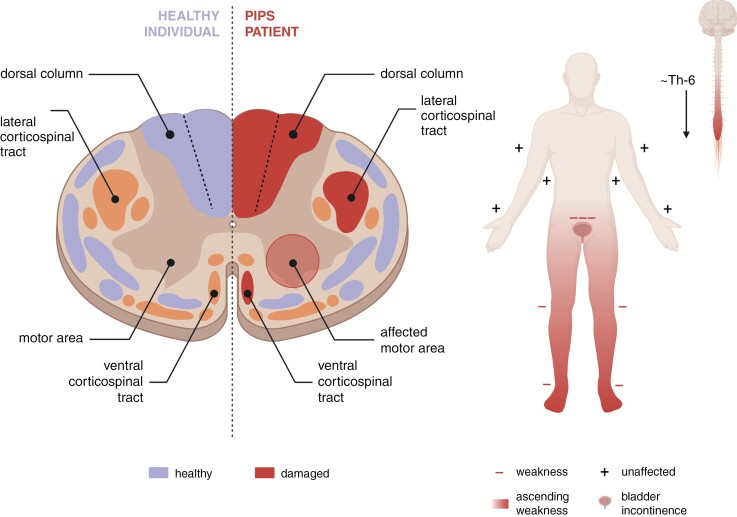
Anatomical distribution of affected areas in PIPS patients

**Key Points**

- Post-intrathecal chemotherapy-related paraplegia syndrome (PIPS) is a diagnosis per exclusion and reversed Trendelenburg and halting of chemotherapy is advised during the diagnostic process.
- Several interventions for PIPS were tried; however, effective therapies to improve the quality of life remain to be investigated.

Intrathecal (IT) chemotherapy stands as a widely utilized modality in the therapeutic approach for hematologic malignancies, serving either prophylactically or as an integral component of primary central nervous system (CNS)-directed regimens. Divergence persists across countries and medical institutions concerning the protocols governing IT therapy administration.^1^ As with all chemotherapeutic interventions, IT therapy carries inherent risks of adverse effects. Of particular concern is the heightened susceptibility of pediatric patients to adverse neurodevelopmental consequences due to chemotherapy-induced interference with neural maturation.^2,3^ Post-chemotherapy side effects are typically attributed directly to the therapeutic regimen; however, the underlying pathophysiological mechanisms often remain obscure.

Ascending paralysis represents notable sequelae of IT chemotherapy, commonly denoted as toxic myelopathy or toxic radiculitis.^4–33^ Diagnostic attribution necessitates the exclusion of alternative etiologies such as Guillain-Barré syndrome (GBS)^20,29^ or CNS metastases. Despite its recognized association with neurotoxicity, comprehensive delineation regarding the onset, clinical manifestations, and therapeutic modalities for IT chemotherapy-related side effects is frequently deficient, potentially impeding timely recognition and management.^34,35^ The fact that these side effects are poorly defined probably causes a delay in recognition and could, therefore, potentially lead to poorer outcomes in these patients. Incidence escalation of ascending paralysis cases within our institution prompted a comprehensive review of hematologic cancer patients experiencing this phenomenon following IT chemotherapy.^36^ Considering the multitude of diagnostic terms employed in literature, we advocate for a unifying nomenclature encapsulating the clinical spectrum: post-intrathecal chemotherapy-related paraplegia syndrome (PIPS). We outline a diagnostic approach tailored to PIPS and underscore the imperative of differentiating it from other syndromes, often erroneously diagnosed and treated with intravenous immunoglobulins (IVIGs) or corticosteroids, seeming ineffective. Our findings suggest nonimmune-mediated pathogenesis for PIPS, warranting exploration of anatomically targeted therapeutic strategies, including reversed Trendelenburg positioning or cerebrospinal fluid (CSF) replacement, to ameliorate its course. We furnish prognostic insights and delineate current therapeutic interventions aimed at mitigating or reversing the trajectory of PIPS.

## Methods

This systematic review complies with Preferred Reporting Items for Systematic Reviews and Meta-Analyses guidelines for individual patient data (IPD).^37^ Before initiation, details of the protocol for this systematic review were registered on the International Prospective Register of Systematic Reviews PROSPERO (CRD42022362121). No funding was acquired for conducting this review.

### Search Strategy and Selection Criteria

Cochrane, Embase, and PubMed databases were systematically searched until March 18, 2021, with search terms “intrathecal” AND “myelopathy OR spinal cord OR paraplegia” AND “cancer OR hematological” (see Supplementary Table S1: p. 1) for a complete list of search terms. The search was performed under supervision of a librarian. Two independent authors (M.M. and G.H.) performed title/abstract screening and selected the studies eligible for full-text screening under supervision (T.F.) using Rayyan (Rayyan Systems).^38^ Any disagreements were resolved through discussion with a hemato-oncologist (T.F.) and neurologist (R.B.). After title/abstract screening, full-text screening and data extraction were independently performed (M.M. and G.H.) and afterward peer-reviewed by T.F. Reference lists of the included articles were manually reviewed for additional articles. Duplicates were filtered with EndNote (Clarivate Analytics). Predefined criteria were set up (M.M., G.H., R.B., G.J.K., and T.F.) and used for inclusion and data extraction to maximize inter-reviewer reliability.

Studies were included if they reported original data of patients with a primary hematological malignancy receiving IT chemotherapy with a subsequent event of ascending paraplegia (eg transverse myelitis and radiculitis) of unknown cause. Other routes of administration, such as ventricular reservoirs, were excluded. A detailed description of the primary hematologic malignancy and concurrent treatments for the initial disease was first extracted, followed by comprehensive data collection on the onset, diagnosis, and treatment of PIPS (eg time of onset, early clinical signs, and blood tests). Non-English articles, in vitro studies, no magnetic resonance imaging (MRI) data, or no full texts were excluded. Diagnoses not attributed to the IT chemotherapy were excluded (eg HIV myelitis). Patients with positive CSF samples (we included patients in complete CNS remission) for malignancy or any microbial disease at the time of onset of the paraplegic event were excluded. There were no restrictions in terms of publication date. The majority of the inclusions are case reports; therefore, no critical appraisal was performed. IPD and aggregated data were both extracted and separately analyzed. Articles that were included, but afterward excluded from data analysis (missing values or unclear data) are marked red (*see*Supplementary Table S3: pp. 3–35). A rerun was performed on February 28, 2023, of which screening and data extraction were independently performed by M.M. and S.C. and peer-reviewed by T.F.

All final diagnoses identified as PIPS were reported and categorized into either myelopathy-like or radiculopathy-like syndromes as described in the included articles. Time of onset was reported as an event after the first IT treatment. If the time of onset was unclear, total IT injections were reported. Figures were made in BioRender.

## Results

**Figure 1.**
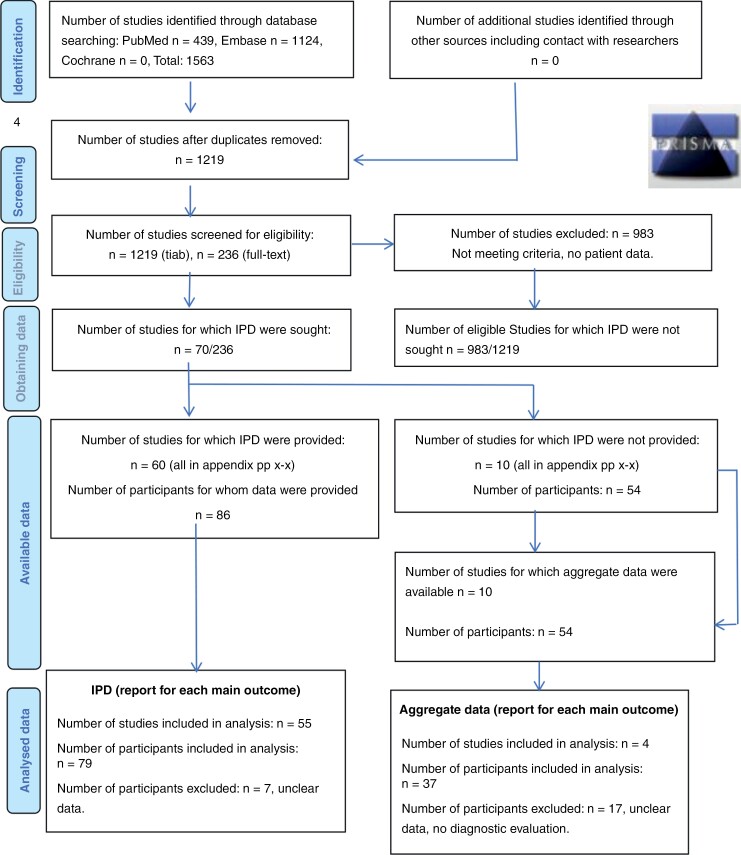
The PRISMA IPD flow diagram.

Out of 1219 articles screened via title and abstract, 236 underwent full-text screening, resulting in 70 being deemed eligible for data extraction due to their documentation of original patient data. Among these, 60 articles provided IPD, whereas 10 articles presented aggregated data. Final analyses encompassed 55 IPD articles (*n* = 79 patients) and 4 articles with aggregated data (*n* = 37 patients), as illustrated in Figure 1. Detailed descriptions of the patients’ primary hematologic malignancy and treatments are shown in Table 1 and Supplementary Tables S2 and S3. Notably, 14 publications with representable cases (6 pediatric, 11 adults, and 3 unclear) were excluded due to missing data, as highlighted in red within Supplementary Table S3.^5,13,22,25,41–50^ The full list of articles and details of final patient inclusions are available in Supplementary Tables S2 and S3.

**Table 1.** Characteristics of the Final 116 Included PIPS Patients

Pediatric: *Individual patient data (n = 32)/Aggregate: (n = 25)*		Adult: *Individual patient data (n = 47)/Aggregate (n = 12)*	
Age	Mean = 10.4 y Range: 1-18	Age	Mean = 44.1 y Range: 21–80
Sex (m/f)	17/14	Sex (m/f)	21/20
Positive CNS status at initial diagnoses/out of total patients	IDP: *N* = 7/25^a^ (5 missing data) Aggregate: *n* = 8/25^a^	Positive CNS status at initial diagnoses/out of total patients	IDP: *N* = 23/47^a^ Aggregate: *n* = 8/12^a^
Primary malignancy	IDP: ALL *n* = 17, AML *n* = 2, Burkitt *n* = 12, CNS Lymphoma *n* = 1 Aggregate: B/T ALL *n* = 15, *ALL n = 8*^b^, Burkitt *n* = 2	Primary malignancy	IDP: ALL *n* = 23, AML *n* = 3, NH *n* = 1, DLBCL *n* = 14, T-LBL *n* = 1, BPD *n* = 1, MALT *n* = 1, TCL *n* = 1, FL *n* = 1, LPL *n* = 1 Aggregate: PCNLS *n* = 8, *ALL n = 4*^b^
MRI results +/total	IDP: myelopathy *n* = 8/23, radiculopathy *n* = 4/9 Aggregate: myelopathy: *n* = 4/7^39^; radiculopathy: *n* = 0/8^39^	MRI results	IDP: myelopathy *n* = 11/30, radiculopathy *n* = 10/17 Aggregate: radiculopathy: 0/8^40^
Time of onset^c^ (1) days after first IT, (2) days after last IT	(1) Median of 14 days, (2) <10 days after their last IT injection (median of 6; range: 3-11) Aggregate: 10 IT, symptoms occurred 13 days after last IT	Time of onset^c^ (1) days after first IT, (2) days after last IT	(1) Median 10.5 days after first IT, (2) 10 days after a median of 6 IT injections Aggregate: second IT and within 5 months after the last injection

Abbreviations: ALL, acute lymphoblastic leukemia; AML, acute myeloid leukemia; NH, non-Hodgkin; DLBCL, diffuse large B cell lymphoma; T-LBL, T cell lymphoblastic leukemia; BPD, blastic plasmacytoid dendritic cell neoplasm; MALT, mucosa-associated lymphoid tissue; TCL, T cell lymphoma; FL, follicular lymphoma; PCNLS, primary CNS lymphoma; LPL, lymph plasmatic lymphoma; IDP, Individual patient data.

^a^All patients were negative for CNS involvement at the time of onset of the neurological symptoms.

^b^This study merged pediatric and adult patients.^39^

^c^Time of onset is set as the starting of symptoms after the first IT injection or as the starting of symptoms after their last injection. The last injection reflects their last injection before the onset of symptoms, not the last IT of a completed cycle.

For analytical purposes, we segregated patients into pediatric (age < 21) and adult (age > 21) cohorts. Final diagnoses in the included articles were predominantly based on clinical manifestations, MRI findings, and laboratory results. Notably, 2 phenotypical diagnoses emerged: a myelopathy-like syndrome and a radiculopathy-like syndrome. These distinct presentations were merged and collectively referred to as PIPS, owing to the ambiguity surrounding the diagnostic criteria and lack of clear differentiation as observed in the included literature.

### Baseline Data

The primary hematologic malignancy and treatment modalities are delineated in Table 1, supplemented by additional details provided in Supplementary Tables S2 and S3. Diagnoses of PIPS were ascertained in adult cases, comprising 29 instances of myelopathy-like and 18 cases of radiculopathy-like presentations, along with pediatric cases comprising 22 myelopathy-like and 9 radiculopathy-like presentations.

All patients enrolled in this study exhibited a negative CSF status for malignant cells at the onset of PIPS manifestation. Evaluation of CNS status preceding PIPS onset revealed that among patients with IPD, 49% of adults (*n* = 23/47) and 30% of pediatric patients (*n* = 7/32) initially tested positive for CSF malignancy at the time of initial diagnosis. In aggregate patient data analysis, 66.7% of adult patients (*n* = 8/12) and 32% of pediatric patients (*n* = 8/25) were initially positive.

In adults, overall mortality not directly linked to PIPS stood at 51% (*n* = 27), with 27% (*n* = 10) observed in pediatric patients. Among deceased adults, 19% (*n* = 5/27) exhibited progressive paralysis leading to respiratory or organ failure, whereas the remaining individuals (*n* = 19) succumbed to causes unrelated to PIPS, including organ failure, gastrointestinal bleeds, intracranial bleeds, infections, or recurrent disease. Among pediatric patients who passed away (9/10), progressive paralysis with brainstem involvement was noted, culminating in respiratory failure.

### Initial Treatment and Onset of Post-IT Paralysis Syndrome

The treatment regimens for patients included triple IT therapy consisting of hydrocortisone, cytarabine/Ara-C, and methotrexate/MTX, or standalone IT treatment involving MTX, hydrocortisone, or Ara-C. Treatment regimens for the primary malignancies were highly variable, and the documentation was often insufficient to compare. Administration of intravenous (IV) nelarabine with IT chemotherapy was observed in 5 cases, which was the only concurrent chemotherapy.^4,18,21,29,39,51^ Despite this, the heterogeneity and ambiguous reporting concerning dosing, timeframe, and administration schedules precluded us from a statistical analysis regarding correlations between chemotherapeutic agents and the onset of events.

In adult patients, the time lapse between the initial IT injection and the manifestation of symptoms was documented in 57% (*n* = 27/47) of cases. Of these, 88% (*n* = 24/27) experienced symptom onset with a median interval of 10.5 days (range: < 0.5 h to 59.6 d) following the first IT chemotherapy injection. In the remaining 3 cases, symptoms surfaced after 3, 7, and 12 months, respectively. Symptom onset in the remaining 20 adult cases was linked to the time after their last IT injection. Among these, 85% (*n* = 17/20) developed symptoms within <11 days post their last IT injection (mean of 6 injections; range: 3-13), whereas timing in 3 patients remained uncertain due to unclear documentation.

In pediatric cases, the interval between the initial IT injection and symptom onset was reported in 22 patients, with 86% (*n* = 19/22) exhibiting symptoms at a median of 14 days (range: <0.1 h to 59.9 d). Three patients experienced symptom onset after 3, 12, and 13 months, respectively. The remaining 10 patients displayed symptoms within <10 days post their last IT injection (median of 6; range: 3-11). Aggregate data encompassed 8 adults and 17 pediatric patients, with symptoms typically arising between the second IT injection and within 5 months post the final injection in adults, whereas pediatric patients, who received a median of 10 IT injections, experienced symptoms at a median of 13 days post the final injection. Pinnix et al. combined pediatric with adult patients and described symptoms occurring after a median time of 15 (3-30) days after the last IT injection.

Collectively, in both adults and children, the majority of PIPS cases emerged within 10 days post an IT injection, with no discernible correlation observed between the number of injections (ranging between 1 and 13), type of treatment, and the onset of symptoms.

### Symptoms

Manifestations in patients afflicted by PIPS typically manifested acutely or progressed gradually over a span of 2 to 14 days subsequent to their initial presentation. Commonly reported symptoms, often occurring concomitantly, included paresthesias in the lower limbs and/or sacral area, muscle weakness, an unsteady gait, or difficulties with urination/defecation. Both unilateral and bilateral symptom onset was reported, characteristically following a progressive and ascending trajectory. Some patients reported prodromal symptoms days or weeks preceding the onset of paralysis, such as sharp shooting pains in the lower limbs, knee, hip, or lower back, accompanied by fevers and chills.

Articles reporting pain as a prominent symptom tended to diagnose a radiculopathy-like syndrome as the final diagnosis. Prodromal symptoms were documented in 11% (*n* = 5) of adult cases, whereas descriptions of such symptoms were absent in pediatric patients. Ascending paralysis typically progressed around the T6-T12 spinal levels (~95% of cases), though in rare instances, it progressed to quadriplegia, with autonomic dysfunction culminating in respiratory failure (~5% of cases).

**Figure 2.**
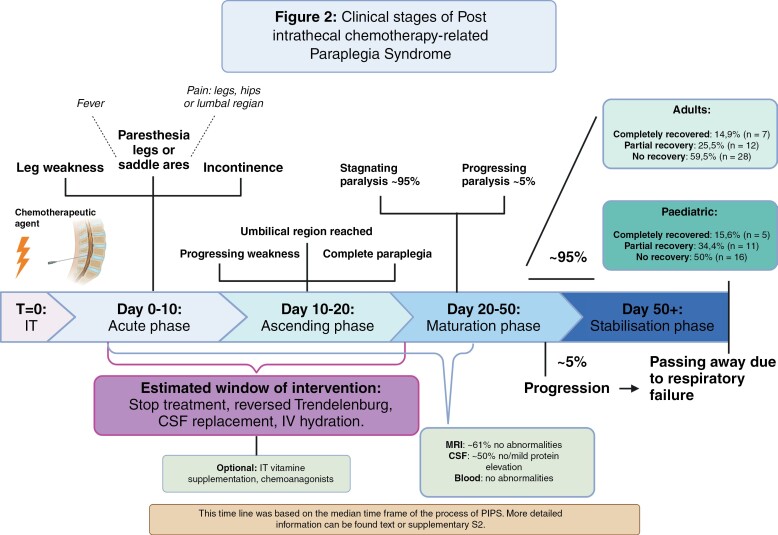
Clinical stages of post-IT chemotherapy-related paraplegia syndrome.

Deep tendon reflexes were frequently absent, and vibration sense was consistently affected, albeit the Babinski sign exhibited variability among patients and occasionally manifested unilaterally. Patients were often rendered unable to walk independently and were reliant on wheelchairs. Neurological examinations commonly revealed pure posterior column syndromes, pure ventral motor syndromes, or mixed syndromes extending up to the T6 level. A graphical depiction of the affected regions is provided in the graphical abstract, whereas clinical staging based on median time frames is illustrated in Figure 2. In cases where disease progression extended beyond T6, patients typically transitioned to complete quadriplegia, often requiring ventilatory support and occasionally progressing to a comatose state.

### MRI, CSF, and Blood Results

Articles initially based their diagnoses on clinical observations, subsequently based on MRI and blood results. Diagnoses by exclusion were characterized as toxic myelopathy or toxic polyradiculopathy-like syndromes, merged under the term PIPS as previously described. Among adult PIPS patients diagnosed with myelopathy-like symptoms, 38% (*n* = 11/29) exhibited pathological MRI findings initially, with 72% (*n* = 21) demonstrating changes upon follow-up (>1 month). Hyperintensity on T2 images, primarily in the dorsal region spanning from T6 to the conus, was prevalent. In adults with radiculopathy-like symptoms, 41% (*n* = 7/17) displayed MRI abnormalities in the ventral roots, with no observed signal escalation over time. In pediatric PIPS patients diagnosed with myelopathy, 36% (*n* = 8/22) exhibited initial MRI pathology, escalating to 54% (*n* = 12/22) over time, mirroring findings in adults. Among pediatric patients diagnosed with radiculopathy, 44% (*n* = 4/9) displayed MRI abnormalities.

A standardized protocol or definitive biomarker for PIPS diagnosis remains elusive. At onset, patients tested negative for viral, bacterial, or parasitic pathogens in blood and CSF samples, with no malignant cells detected in any CSF specimens. Approximately 50% of patients exhibited mild elevation in CSF protein, occasionally increasing over time. Although the majority tested negative for myelin basic protein (MBP), some showed elevated levels upon follow-up. Vitamin deficiencies were uncommon. A summary of commonly utilized diagnostic tests reported in the literature and their comparison with PIPS is provided in Supplementary Table S4.

### Intervention

Efforts to manage PIPS predominantly involved steroid administration. However, alternative interventions such as IVIG, vitamin B12 or folic acid supplementation, various resting positions (eg reversed Trendelenburg), CSF replacement, or discontinuation of chemotherapy were also explored. Given the inherent difficulty in directly comparing these diverse treatments, we aggregated the outcomes.

**Figure 3.**
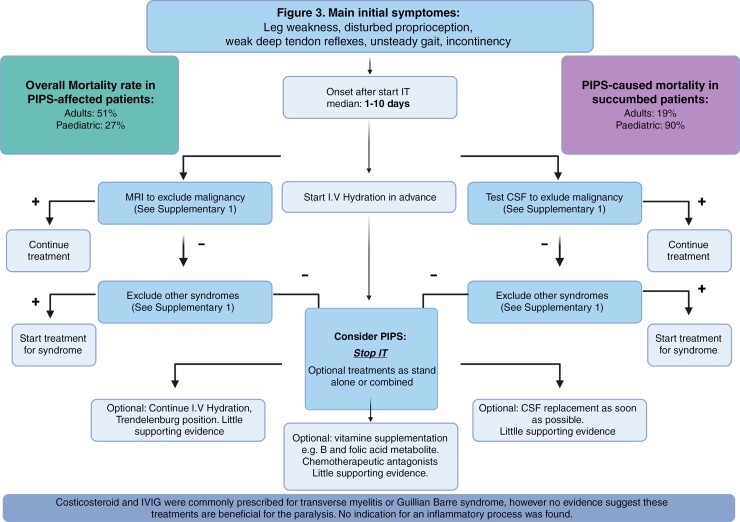
Decision chart.

To assess the potential benefit of any treatment versus no treatment, patients were stratified into 3 subgroups: (1) completely recovered, (2) showing improvement, or (3) exhibiting no recovery. Each subgroup was further divided into treated and non-treated categories. Among adults, 85% (*n* = 6/7) of those completely recovered underwent treatment compared with 58% (*n* = 7/12) in the improvement group and 57% (*n* = 12/28) in the no recovery group (Fisher exact test: *P* = .1179). In pediatric patients, 60% (*n* = 3/5) in the completely recovered group received treatment, as did 36% (*n* = 4/11) in the improvement group and 63% (*n* = 10/16) in the no recovery group (Fisher exact test: *P* = .7157). These findings suggest no significant difference in recovery outcomes related to treatment, indicating that within this dataset, treatment does not appear to predict improvement or recovery from PIPS. Aggregate data revealed no instances of improvement among patients. A decision tree detailing these outcomes is provided in Figure 3.

### Autopsy Reports

Seven autopsy reports were identified^19,22,52–56^ with one excluded from the final analysis due to incomplete data.^22^ The reports collectively depict alterations localized to the white matter, characterized by extensive necrosis in the lower spinal region, fibrillary gliosis, demyelination, and axonal degeneration. Notably, changes were most pronounced in the dorsal columns. More cranially, vacuolar degeneration was observed in the spine. While 2 autopsies revealed the presence of macrophages,^52,55^ the remaining reports did not exhibit signs of immune infiltrates.

## Discussion

The incorporation of IT chemotherapy is integral to numerous treatment regimens for hematological malignancies, either as a preventive measure or for addressing CNS involvement. This systematic review delineates the profiles of 116 hematological cancer patients afflicted by sudden paraplegia subsequent to IT chemotherapy. Although over 140 patients were identified, some were excluded due to incomplete data, possibly indicating an underestimation of the actual frequency of PIPS. Not all clinicians may recognize or report this syndrome, further suggesting that our dataset may underestimate the true frequency. Articles relied on clinical observations, MRI findings, and CSF/blood analyses for diagnoses, yet significant variability existed in approaches to this phenotypically ambiguous ascending paraplegia. While diagnoses were often concluded as myelopathy- or radiculopathy-like syndromes, caution was exercised in definitive statements. Original patient data were extracted from case reports and trial studies, which exhibited heterogeneity in data documentation.

All patients presented with negative CSF findings for malignancy at the onset of PIPS, indicating the absence of cancer cell involvement. While initial CNS engagement was observed in less than half of the cases, the possibility of PIPS serving as a secondary cause of CNS impairment cannot be entirely discounted. Mortality rates were notably higher among adults (51%) compared with pediatric patients (27%), consistent with trends in cancer treatment-related outcomes.^57^ Notably, PIPS-related mortality reached 18.5% in adults versus a staggering 90% in pediatric cases, potentially attributed to diagnostic challenges in the younger patients and subsequent delays in intervention leading to respiratory failure. Additionally, uncertainty remains if there are pathophysiological differences between the underlying mechanisms of PIPS in pediatric and adult patients. As the nervous system is still developing in pediatric patients, PIPS may manifest more drastically in an all-or-none fashion.

The initial therapeutic approach for cancer entailed either triple IT therapy comprising hydrocortisone, Ara-C, and MTX, or standalone IT regimens, occasionally complemented by IV nelarabine administration. Given the diversity in treatment modalities, discerning the singular impact of any particular agent on PIPS remains elusive. Additionally, the contribution of nelarabine in the development of PIPS remains indeterminate. It is plausible that nelarabine may predispose patients to PIPS, or that specific combinations of agents may heighten the risk. Notably, only a small subset of patients—5 in total—received nelarabine treatment, suggesting that other factors may predominantly underlie the development of PIPS.

The determination of onset time has been delineated in relation to either the first or last IT injection. However, considering that cessation of treatment typically coincides with the manifestation of PIPS symptoms, the timing of the last IT injection may not necessarily signify the conclusion of the treatment regimen. Evidently, PIPS tends to manifest approximately 1-10 days postinjection, irrespective of the cumulative number of prior administrations. While intuitively linked to IT injections, the etiology of PIPS appears multifactorial, influenced by various confounding and physiological factors. It is conceivable that the metabolic status of patients may predispose them to PIPS following IT administration.^58^

The diagnostic process for PIPS is often protracted due to the necessity of excluding alternative diagnoses such as GBS, transverse myelitis, radiculitis, or vincristine intoxication. These conditions typically take precedence and must be meticulously ruled out before arriving at a conclusive diagnosis. In the literature, final diagnoses have commonly been denoted as toxic myelopathy or radiculopathy, transverse myelitis, or methotrexate (MTX) toxicity.

To streamline diagnostic clarity and reflect the multifaceted nature of the syndrome, we advocate for the adoption of the term PIPS. This terminology encapsulates the amalgamation of symptoms observed in affected patients, which often encompass a spectrum of clinical presentations overlapping with the aforementioned syndromes. Omitting terms like “myelitis” or “radiculitis” is warranted as there is scant evidence supporting inflammatory processes, as evidenced by the lack of response to steroids and autopsy findings indicative of minimal immune infiltration.

PIPS can potentially progress to involve the brainstem, posing a lethal threat. However, in the majority of cases, the syndrome halts its progression at a specific thoracic level, resulting in lower limb paralysis that significantly impacts the patient’s quality of life. This delineation underscores the varied clinical course and potential outcomes associated with PIPS, emphasizing the need for accurate diagnosis and comprehensive management strategies.

Regrettably, specific diagnostic markers for PIPS remain elusive. Blood and CSF analyses yielded predominantly negative results, as detailed in Supplementary Tables S2 and S3. While some patients exhibited a mild increase in CSF total protein and MBP levels, the majority presented with normal findings. Investigations did not reveal evidence of infections or autoimmune etiologies. Additionally, levels of vitamin B and folic acid were within normal ranges for most patients. MRI findings indicated initial lesions in approximately 40% of patients with presentations resembling myelopathy, a proportion that escalated to 70% over time. However, it is crucial to note that negative MRI results did not preclude the possibility of PIPS. Unfortunately, negative MRI findings are sometimes misconstrued as evidence against PIPS, leading to delays in intervention and potentially permitting the progression of PIPS to an irreversible state. Among PIPS patients exhibiting symptoms akin to radiculopathy, approximately 50% displayed heightened signals in the ventral roots, which remained consistent over time. These observations underscore the necessity of approaching PIPS diagnosis through a process of exclusion. We should be cautious by ruling out PIPS solely on negative MRI, CSF, or blood samples. Therefore, we advocate for a cautious approach whereby further IT is deferred pending a comprehensive work-up aimed at identifying alternative causative factors.

Treatment strategies for intervening in PIPS have exhibited considerable heterogeneity, encompassing a range of modalities such as cessation of treatment, administration of anti-inflammatory agents (eg steroids and IVIG), vitamin supplementation (eg B12 and folic acid), dextromethorphan, adoption of reversed Trendelenburg positions, and CSF replacement. We could not find a difference in recovery between the treated and untreated groups. We acknowledge that we did not prespecify the end points, as we were unsure of what to expect based on the literature. This approach makes performing statistics highly susceptible to skewing results in any direction.

Although some studies have reported favorable outcomes with steroid therapy,^12,32,59,60^ others have disputed its efficacy in halting or reversing PIPS progression. Despite the minority suggesting a beneficial effect of steroids, the prevailing consensus among most articles is that steroids do not confer significant benefits in managing PIPS. Similarly, interventions involving IVIG, dextromethorphan, and vitamin replacement have failed to demonstrate improvement in patient outcomes, even when administered promptly following symptom onset. Notably, anecdotal reports have highlighted instances of successful resolution following unconventional interventions. For instance, one patient underwent CSF replacement and exhibited remarkable improvement, regaining ambulatory function within 3 days, with complete resolution of PIPS observed after 3 months.^26^ By replacing the CSF, you can quickly eliminate the chemotherapy, potentially halting the progression. If the metabolic state contributes to PIPS development, removing the metabolic waste products might be beneficial for the patient. In another case, a patient placed in the reversed Trendelenburg position and administered IV hydration alongside hydrocortisone experienced a rapid descent in sensory deficits, with complete resolution of myelopathy evident within 17 h of symptom onset, devoid of residual sequelae.^61^ Placing patients in the reversed Trendelenburg position was likely an attempt to prevent the progression of ascending paralysis. However, one could argue that this position may maintain higher drug concentrations in the lower body, potentially causing more local nerve damage. The regular Trendelenburg position could help dilute the drug throughout the CSF possibly protecting the nerves in the lower body. On the other hand, reversed Trendelenburg could increase the blood flow to the lower part of the CNS, possibly having a protective effect. This patient also received hyperhydration, which might play a significant role in mitigating the ascending paraplegia. Although spontaneous improvements have been observed in a select few adult patients following discontinuation of IT treatment, this cessation did not universally arrest the progression of PIPS in most cases. Thus, the optimal management approach for PIPS remains elusive, warranting further research to elucidate effective therapeutic strategies.

In conclusion, PIPS presents a diagnostic challenge, often overlooked or misattributed during clinical assessment as either immune-related or toxicity-induced. Steroids, a commonly employed intervention, have shown limited efficacy in mitigating PIPS progression among the majority of patients. While anecdotal evidence suggests potential benefits of interventions such as CSF replacement, drainage, lavage, hyperhydration, and the reversed Trendelenburg position in select cases, further research is needed to ascertain their therapeutic value in delaying or reversing PIPS.^62^ Despite categorizing ascending paraplegic syndromes under the umbrella of PIPS due to shared clinical features, distinctions may exist between myelopathy- and radiculopathy-like presentations, suggesting potentially divergent pathophysiological mechanisms. The under-recognition of PIPS may underestimate its frequency, impacting patient quality of life significantly. Diagnosis relies on comprehensive clinical and diagnostic evaluations, acknowledging that negative MRI and blood results do not preclude PIPS diagnosis. A crucial recommendation is the immediate cessation of IT chemotherapy upon PIPS symptom onset, followed by a thorough exploration of alternative etiologies for the paraplegic syndrome. Our patient population suggests that discontinuing IT chemotherapy does not pose a significant risk of cancer recurrence, with cases reported to remain cancer-free post-cessation. However, this consideration applies only to patients with clear CSF, as our inclusion criteria required the absence of malignant cells in the CSF. Additionally, not all patients were followed for the remainder of their lives, making it difficult to definitively state that tumor recurrence would not occur. We still recommend rechallenging patients with chemotherapy upon recurrence of the malignancy. It remains unclear if patients who have experienced and/or are in the stabilized phase are more likely to develop PIPS again after rechallenging. While the efficacy of interventions like the reversed Trendelenburg position in delaying or reversing PIPS is equivocal, their minimally invasive nature warrants consideration, particularly in the early stages of the syndrome. Given our focus on hematological malignancies, it is conceivable that PIPS may also manifest in patients with other malignancies, such as solid tumors and patients with and without active leptomeningeal cancers. At our institution, PIPS occurred exclusively in hematological patients receiving IT therapy, leading us to exclude solid tumors and other administration routes. However, we emphasize the need for heightened awareness and meticulous documentation by clinicians for both hematological and solid tumors. Further research into PIPS holds promise not only for enhancing treatment outcomes but also for uncovering novel mechanisms underlying neural regeneration and recovery. Thus, concerted efforts are warranted to deepen our understanding of PIPS and advance therapeutic strategies to alleviate its burden on affected individuals. Observing the similarities among patients, PIPS appears to progress in an ascending, domino-like fashion. We can only speculate that interrupting the falling tiles could halt PIPS and potentially reverse the damage.

## Supplementary Material

vdae217_suppl_Supplementary_Materials
