# Supplementary material for: Post-intrathecal chemotherapy-related paraplegia syndrome in hematological cancer patients: A systematic review
Source: Neurooncol Adv. 2024 Dec 20;7(1):vdae217. doi: 10.1093/noajnl/vdae217 (PMC11713023; doi:10.1093/noajnl/vdae217)
Supplement: vdae217_suppl_Supplementary_Materials [file vdae217_suppl_supplementary_materials.docx]

**Supplementary**

**Table S1:** Search terms

*PubMed search terms:*

(("Injections, Spinal"[Mesh] OR intrathecal*[tiab] OR intraspinal*[tiab]))

**AND**

((Paralysis[Mesh] OR Paresis[Mesh] OR Spinal Cord Diseases[Mesh] OR "Myelitis, Transverse"[Mesh] OR transverse myel*[tiab] OR myelitis*[tiab] OR myelopath*[tiab] OR paraplegia*[tiab] OR paralys*[tiab] OR pares*[tiab] OR "Polyradiculoneuropathy"[Mesh] OR "Neurotoxicity Syndromes"[Mesh] OR polyradiculo*[tiab] OR neurotoxicit*[tiab] OR "Radiculopathy"[Mesh] OR radiculopath*[tiab] OR radiculitis*[tiab]))

**AND**

("Leukemia"[Mesh] OR "lymphoma"[Mesh] OR "Hematologic Neoplasms"[Mesh] OR leukemi*[tiab] OR leukaemi*[tiab] OR lymphom*[tiab] OR lymfom*[tiab])

*Embase (Elsevier) search terms:*

('intraspinal drug administration'/exp OR intraspinal*:ti,ab,kw OR intrathecal*:ti,ab,kw)

**AND**

'spinal cord disease'/exp OR 'paralysis'/exp OR 'paresis'/exp OR myelopath*:ti,ab,kw OR paralys*:ti,ab,kw OR pares*:ti,ab,kw OR 'transverse myel*':ti,ab,kw OR myelitis*:ti,ab,kw OR paraplegia*:ti,ab,kw OR 'neuropathy'/exp OR 'neuropath*':ti,ab,kw OR 'neurotoxicity'/exp OR 'neurotoxicit*':ti,ab,kw OR 'radiculopathy'/exp OR 'radiculopath*':ti,ab,kw OR myeloradiculopathy*:ti,ab,kw OR 'delayed neurotoxicity'/exp OR polyradiculoneuropathy:ti,ab,kw

**AND**

('hematologic malignancy'/exp OR lymphom*:ti,ab,kw OR lymfom*:ti,ab,kw OR leukemi*:ti,ab,kw OR leukaemi*:ti,ab,kw OR 'blood cancer cell line'/exp OR 'lymphoproliferative disease'/exp OR 'lymphatic system malignancy'/exp)

**AND** 'conference abstract'/it)

**Table S2:** Included articles and main outcome:

| **Reference** | **Primary malignancy (CNS: +/-)** | **Initial treatment** | **Symptoms** | **Treatment outcome** |
| --- | --- | --- | --- | --- |
| Alsdorf et al. (2015)^33^ | DLBCL, CNS - | R-CHOP, R-DHAP, TIT. | 2d after triple IT: nausea, vomiting, 2 days later rapidly ascending, irreversible sensory, motor, and autonomic dysfunction and paraplegic. | HD methylprednisolone: no improvement. Remained stable. |
| Amer-Selas et al. (2021)^23^ | T-ALL, CNS - | Nelarabine, prophylactic TIT. | After 30d: dysesthesias lower limbs, leading to paraplegia, paresis of upper limbs and urine retention and constipation. | Vitamin B6 and B12, high dose, dexamethasone. 4 times plasma exchange. No improvement. Paraplegic after ITT and nelarabine. |
| Anderson et al. (2002)^64^ | ALL, CNS- | IT MTX, Ara-C, and hydrocortisone, I.V. vincristine, L-asparaginase, cyclophosphamide, oral MTX, and 6-mercaptopurine. | 12d after starting IT: lower extremity flaccid weakness, inability to walk, and mild upper extremity weakness over a 2-week period, absent patellar and ankle tendon reflexes, normal sensation. | IVIG and within 3 days proximal leg strength improved. Ventral polyradiculopathy after IT. Improvement after 4 months and able to walk again. |
| Ateşoǧlu et al. (2007)^65^ | ALL-L1 (pre-B), CNS - | Hyper-CVAD. IT MTX. | 2d after IT MTX: unable to urinate for nine hours. Constipated for six days. | IT stopped. After 21 days regained complete function of bladder and bowel. |
| Bellon et al. (1995)^66^ | Burkitt Lymphoma | Induction IT MTX Ara-C (hydrocortisone vincristine, cyclophosphamide, mesna, Adriamycin (doxorubicin), and methylprednisolone. Consolidation with I.V. VP-16 (etoposide) and ifosfamide. 1800 cGy cranial to C3. | 24h after last IT (within 5.5 weeks): paresthesia’s, numbness, weakness of both lower limbs. 2d later unable to walk. Ascending paraparesis, 2/5 muscle strength in the lower extremities. Diffuse numbness to the nipple level and incontinence of urine. By day 79: complete quadriplegic. | Dexamethasone. No improvement. Diffuse polyradiculopathy into quadriplegia. No improvement after steroids. Died of respiratory failure. |
| Breuer et al. (1977)^67^ | AML, CNS- | VAPA chemotherapy (vincristine, Adriamycin, prednisone, cytosine arabinoside. CNS prophylaxis: 6x IT Ara-C. Concurrent whole brain irradiation (2550 rads). | 2w after last dose of IT: instability of gait, weakness of lower extremities and urinary incontinence. Broad-based gait with normal cerebellar function, diffuse weakness of the legs both proximally and distally, marked position sense loss, sense loss to the hips, diminished touch, and position sense distally, a positive Romberg, hyperactive knee jerks, absent ankle jerks and bilateral extensor plantars. | Paraplegia after IT. Marked improvement over time (3 months) and able to walk. Died of GI bleeding. |
| Byrnes et al. (2019)^68^ | DLBCL, CNS- | Rituximab, dexamethasone, high-dose cytarabine, and cisplatin (R-DHAP). IT MTX prophylactically in each cycle. | 1 day after first IT MTX and cytarabine: non-positional headache, photophobia, nausea without vomiting, and double vision, rectal incontinence, and urinary retention. Weakness progressed to bilateral lower extremity paralysis. Diminished reflexes in the bilateral patellar and Achilles tendons, positive Babinski on the right, and diminished sensation to light touch. | Polyradiculitis, no recovery, progressed soon and passed away due to systemic lymphoma. |
| Cachia et al. (2015)^19^ | **1pt:** 70y, B-ALL Ph-  **2pt:** 59y, B-ALL Ph-  **3pt:** 18y, Burkitt, CNS relapse  **4pt:** 32y, B-ALL Ph-, CNS relapse  **5pt:** 48y, B-ALL, relapse  **6pt:** 50y, CML Ph+, CNS relapse  **7pt:** 35y, B-ALL Ph-, CNS relapse | **1pt:** Hyper-CVAD, rituximab, inotuzamab, ozogamycin **2pt:** Hyper-CVAD, Ofatumumab **3pt:** Rituximab, hyper-CVAD, anti CD19 Ab: MOR0028. **4pt:** Hyper-CVAD, everolimus, clofarabine, idarubicin, cytarabine, rituximab, vincristine, anti CD19 Ab. 92 cycles: MOR0028. **5pt:** CALGB, CD19 Ab Maytansine conjugate, Hyper-CVAD, inotuzamab ozogamycin. **6pt:** Imatinib, Allogenic stem cell with cyclophosphamide. **7pt**: Hyper-CVAD, POMP maintenance, Augmented hyper-CVAD. | **1pt**: Dexamethasone, dextromethorphan, IVIG 5 days. **2pt:** dexamethasone, IVIG x 5 days, Vitamin B12. **3pt:** Dexamethasone, Vit B12, IVIG 95 days. **4pt:** Dexamethasone, folic acid, CSRT. **5pt:** IVIG x 5 days. **6pt:** CSRT. **7pt:** Pregabalin, dextromethorphan. | **1pt:** Neurological improvement but  died 1 month organ failure.  **2pt:** No neurological improvement.  Alive at 6 months post onset symptoms.  **3pt:** No neurological  improvement. Died  5 weeks after onset from organ failure.  **4pt:** No neurological  improvement. Died  2 months post onset.  **5pt:** No neurological improvement. Died  2 months post onset.  **6pt:** No neurological improvement 6 months post onset. Died  7 months post onset.  **7pt:** Neurological improvement post rehab but developed LMD 2 months later with neurological deterioration. |
| Castillo-Torres et al. (2020)^26^ | ALL | I.V doxorubicin and vincristine, and IT MTX, ARA-C and dexamethasone. | During 5^th^ IT: dizziness and headache, and after the administration lost consciousness. On regaining consciousness, he was completely deaf, paraplegic with abolished sensory modalities up to his chest and unable to control sphincters. | Prednisone without improvement. At follow-up 1 month later, strength had improved to 3/5, but patient was unable to weight bare and sphincter function was unchanged. |
| Chukwu et al. (2015)^9^ | ALL | Cyclophosphamide, vincristine (oncovin), cytosine arabinoside and oral prednisolone. IT cytosine arabinoside. | After 4th IT, urine, and fecal retention. After 2 months: restarted and he complained of weakness and tingling sensation in the lower limbs with flaccid paraplegia. Sensation normal. | After first event: chemotherapy withdrawn. She became quadriplegic in 3 days. She had generalized convulsions with labored breathing and died a few days later. |
| Cyriac et al. (2008)^69^ | ALL | BFM 86 protocol. | Quadriparesis within 14 days after IT. | Recovered within 72 hours. |
| Dornbos et al. (2019)^54^ | Blastic plasmacytoid dendritic cell neoplasm, relapse with CNS metastases, ALL. | 5 doses IT cytarabine initial treatment. 7 doses IT cytarabine, MTX, and hydrocortisone with relapse. | 1y post IT: Progressive weakness and paresthesia of the lower extremities, and disturbed vibration and proprioception. Bladder and bowel dysfunction. | Folic acid and cyanocobalamin. Quadriplegic, passed away. |
| Dufourg et al. (2007)^51^ | B/T ALL, n=15 | TIT methylprednisolone (MP), MTX and Cytarabine adjusted for age. | **Spinal involvement** **n=7**; 2/7pt flaccid quadriplegia, sensory and sphincter disorders. 5/7 patients solely motor involvement; paraplegia in three cases with pyramidal signs (Babinski sign, brisk osteo-tendinous reflexes).  **Anterior horn involvement n=3:** Rapidly progressive purely motor neuropathy (flaccid paraplegia).  **Peripheral involvement n=5:** Sensory-motor-radiculo-neuritis, two during consolidation, seven during intensification and five during maintenance therapy. The median TIT number was 10 (2–18), and onset occurred a median of 13 days after the last injection. | 6/15 children fully recovered. 9/15 had chronic sequels and recovered very slowly. Spinal involvement had the worst functional prognosis. 8/9 children showed persistent paraplegia and need for urinary drainage. |
| Dunton et al. (1986)^55^ | AML, CNS-, 2pt. | Protocol #8101. IT MTX, hydrocortisone and Ara-C. Cranial RTX (12x200rad). No spinal radiation. | **Pt 1:** 4m after IT chemotherapy: developed hypesthesia and weakness of the lower extremities, urinary and fecal incontinence.  **Pt 2**: for 2w left knee pain, 4 days of left leg weakness and right foot numbness, and early morning urinary retention. Within 1 month, neck pain, increasing left knee pain, worsening left hemiparesis, and frequent urinary incontinence. | **Pt1/Pt2:** Dexamethasone without appreciable symptomatic improvement.  Chemotherapy was discontinued. Passed away due progressive disease. |
| Gagliano et al. (1976)^70^ | ALL, CNS + | Doxorubicin, vincristine, cytosine arabinoside, and dexamethasone, IT MTX. 2500 rad cranium. | After 8th injection, paraplegia and atonic bladder. | After 8th injection, paraplegia and atonic bladder. 6 weeks after onset: return of deep tendon reflexes, complete left leg function and partially right leg function. |
| Gallego Perez-Larraya et al. (2011)^71^ | **1pt:** DLBCL  **2pt:** MALT  **3pt:** DLBCL  **4pt:** DLBCL | **1pt:** 4 (R-MegaCHOP93, R-CHOP91), IT courses before event: 4  **2pt:** 6 (R-FMD), IT courses before event: 6,  **3pt:** 11 (R-CHOP98, R-ICE93), IT courses before event: 3  **4pt:** 2 (R-Mega CHOP), IT courses before event. | **1pt:** 24d after starts: Conus medullaris/cauda equina syndrome.  **2pt:** 24 days after start: Conus medullaris/cauda equina syndrome.  **3pt:** 24 days after start: Conus medullaris/cauda equina syndrome.  **4pt:** 6 days after start: Conus medullaris/cauda equina syndrome. | **1pt:** none, complete resolution.  **2pt:** 7 days dexamethasone, slight improvement.  **3pt:** none. Slight improvement.  **4pt:** IT withdrawn, complete resolution. |
| Garcia-Tena et al. 1995)^72^ | ALL, CNS - | Vincristine, daunorubicin, prednisone, L-asparaginase, and two doses of TIT, achieving complete remission. LLA-VII-9 1 therapy protocol. | 12h after the last lumbar puncture: severe headache, backache, opisthotonos, and fever. 10 days later: paraparesis, urinary incontinence, and diplopia. | Flaccid quadriplegia with respiratory failure and bulbar involvement (facial diplegia, dysphagia, diplopia with normal ocular motility High-dose I.V. steroids, IVIG, and folic acid were administered without clinical response. |
| Geissler et al. (1997)^73^ | Burkitt Lymphoma | I.V chemotherapy: cyclophosphamide, vincristine, MTX, ifosfamide, prednisolone. IT Ara-C, MTX, and dexamethasone. | Severe central neuropathy within 24h after the first IT injection. 8h post IT, weakness and paresthesia of both legs, urinary retention. After 3d: vomiting, dizziness, and disorientation. Symptoms progressed, tongue weakness, diminished facial sensations and ascending paresthesia up to Th-2. Became complete quadriplegic. | Stopped IT. After 10 days, complete quadriplegia and dependent on respiratory ventilator. Patient passed away without recurrence of the tumor. |
| Gosavi et al. (2013)^22^ | ALL, CNS - | Hyper-CVAD with IT MTX and cytarabine. | 5w after her first dose of IT MTX: numbness in both lower limbs, weakness, areflexic paraparesis, impaired vibration sense in her toes and a positive Romberg’s sign. Second dose days later: no function in the lower limbs, sensory level at T10 and bilateral extensor plantar responses. | **First dose:** She received IVIG for presumptive Guillain–Barré syndrome.  **After second event:** folic acid supplements. She also received five days of IV methylprednisolone. No effects. Complete paraplegia with bladder dysfunction. |
| Hilgendorf et al. (2008)^60^ | **1pt**: c-ALL  **2pt:** AML | IT liposomal cytarabine and oral dexamethasone. GMALL 7/03. | **1pt:** 4 weeks after the last IT dose. (Total 9 doses)  **2pt:** 7 days after first IT dose. | **1pt:** cauda equina syndrome. Remission. No treatment  **2pt**: paraplegia but resolved after dexamethasone, progressive disease. |
| Jabbour et al. (2016)^61^ | Pre-B ALL | Induction hyper-CVAD, completing all 6 doses of liposomal cytarabine. | Developed slow onset of fecal incontinence, urinary hesitancy, and perineal numbness, progressing after cycle 4 of MTX and cytarabine. | Corticosteroids, mild improvement. |
| Joseph et al. (2014)^20^ | Female, 42y, T-ALL. **Relapse:** mediastinal and bone marrow relapse, CSF positive | Hyper-CVAD + 8 cycles IT MTX and cytarabine. **Relapse:** Nelarabine and 2 IT cycles MTX + cytarabine + Hydrocortisone. | **Relapse:** 24 h after last dose, ascending bilateral limb numbness, progressed but stopped at the lower cervical region. | Methyl prednisone without improvement. Complete dorsal myelopathy and loss of proprioception. |
| Kleinschmidt-Demasters et al. (1992)^56^ | NHL High grade, T-cell, immunoblastic | IT Ara-C, IV doxorubicin, cisplatin, and Ara-C. | 8h after induction, hypoxia, widened arterial-alveolar gradient, tachycardia. 30h after induction, blurred vision progressed to total blindness and paraplegia, sensory level T4. Weakness of both arms, followed by coma and unreactive dilated pupils. Flaccid tone in all extremities Deep tendon reflexes absent in arms and legs after 48h. | Ascending quadriplegia up to the medulla oblongata. Extensive transverse coagulative necrosis with neither inflammatory nor macrophage reaction. |
| Koh et al. (1999)^34^ | **1pt**: ALL, CNS prophylaxis,  **2pt**: ALL, CNS Prophylaxis.  **3pt**: ALL, CNS prophylaxis | **Systemic**: 6-mercaptopurine, vincristine, dexamethasone in **1pt** and **2pt**, l-asparaginase, vincristine, prednisone in **3pt**. **IT: 1pt:** IT MTX.  **2pt**: IT MTX, **3pt:** IT MTX. | **1pt:** bilateral leg weakness 2d after 3rd IT MTX, progressive, unable to walk, reflexes and sensory normal. Incontinence.  **2pt**: frequent falls after 11^th^ IT MTX, areflexia and muscle weakness, sensory normal. **3pt:** leg weakness after last IT MTX, unable to walk. Areflexia. | **1pt:** 3 days methyl prednisone IV. Complete recovery after 2 months.  **2pt**: 3 days methyl prednisone IV. Recovered quickly after treatment.  **3pt:** 3 days methyl prednisone IV. Mild improvement, able to stand, but legs remained spastic. |
| Lakhani et al. (1986)^74^ | Ph+ ALL, Relapse patient. | First relapse: IT MTX and Ara-C. Second relapse: IT MTX and Ara-C and hydrocortisone. Resistant disease: IT Mitoxantrone 3 times. | After 2^nd^ IT mitoxantrone: local pain. 3rd dose: again, local pain. 3-4 days later: severe pain in legs, paraplegia within 2 weeks. Absent leg reflexes, no sensory loss or alteration in bowel or bladder function. | Complete paraplegic within 2 weeks of mitoxantrone without sensory problems, bowel, or bladder involvement. Stayed in the chronic phase. |
| Lalayanni et al. (2017)^31^ | T-LBL | CVAD (cytarabine, etoposide, MTX, L-Asparaginase, doxorubicin, vincristine. prophylaxis TIT. Oral 6-mercaptopurine. Nelarabine I.V. | After the 4^th^ nelarabine gift: ascending sensory problems starting in the legs to the anterior abdominal wall, muscle weakness within 10 days. After IVIG, continued to ascend to upper limbs. | **Day 10**: IVIG. **Day 33**: IV high dose steroids. **Day 40**: vitamin supplementation and plasma exchange. Complete paraplegia, bilateral plantar extensor reflex, urinary and bowel dysfunction. Hypoesthesia stabilized at C5-C6. Regained complete function after a year of rehab therapy. |
| Lapucci et al. (2018)^32^ | T-ALL, CNS - | TIT, consisting of cytarabine, MTX, dexamethasone, CNS relapse: liposomal cytarabine. TIT restarted prophylaxes. | 4d after the last TIT (14 x + Ara-C): Incontinence, rapidly progressing ataxic gait, and lower limb hypoesthesia below Th10 level and complete paraplegia up to T4 within 10d. | B12 and folic acid. Plasma exchange and steroids. Complete paraplegia with urinary and bowel involvement, with MRI changes up to the cervical tract. No improvement after therapy. |
| Lee et al. (2008)^75^ | B-ALL, CNS - | VPDL regimen: IV vincristine, prednisolone, I.V. daunorubicin, IM L-asparaginase. On day 42, complete remission. Day 43: IT MTX, cytarabine, and hydrocortisone. | Day 47: ascending leg weakness, unable to walk. No tendon reflexes, mild sensory deficit. Urinary retention. | 3d methyl prednisone. Irreversible paraplegia: Myelopathy or ventral neuropathy with urinary retention. |
| Lewis et al. (2019)^46^ | T-cell lymphoma, CNS + | HCVAD/MA, IT MTX and cytarabine. Second line treatment: gemcitabine and vinorelbine. Third line treatment: ICE (ifosphamide, carboplatin, etoposide Switched to pralatrexate. **During the second event**: IT MTX and cytarabine. | **First event:** After 3rd pralatrexate: bilateral flaccid lower limb paralysis  (Power 0/5), bilateral upper limb weakness (power 3/5), areflexia, a  T7/8 sensory level, and bilateral loss of dorsal column function in her feet.  Progressive tetraplegia. **Second event:** (after restarting treatment) re-presented with bilateral flaccid lower limb paralysis (power 0/5), bilateral upper limb weakness (power 3/5), areflexia, a T7/8 sensory level, and bilateral loss of dorsal column function in her feet. | **First event:** conservative treatment resolved the problem. Ascending paraplegia into tetraplegia. Passed away within two weeks of the events. |
| Lu et al. (2007)^29^ | **1pt:** T-LBL, CNS -  **2pt**: FL, CNS + | **1pt:** IT MTX, Ara-C, and hydrocortisone.  **2pt:** MTX, Ara-C, and hydrocortisone. | **1pt**: weakness of bilateral lower extremities, paresthesia, and voiding difficulty gradually developed. Proprioception severely impaired. Urinary and stool incontinence. **2pt:** bilateral weakness in the lower legs. Sensory impairment in the loves-stock pattern, and severe impairment proprioception. Urinary incontinence. | **1pt:** B12 and folic supplements. **2pt:** nothing. Ascending paraplegia without improvement. MRI changes were almost identical. Both passed away, but not as a consequence of the event. |
| Maramatton et al. (2016)^13^ | CNS DLBCL | Rituximab, MTX, procarbazine, and vincristine; triple IT therapy MTX, cytarabine, and hydrocortisone. | 1w after last cycle of IT: tingling and numbness of both lower limbs into quadriplegia. Difficulty walking, unsteady gait, and a girdle sensation around lower chest bilaterally. Impaired proprioception lower limbs with sluggish deep tendon reflexes and extensor planters. | Injection methyl cobalamin, folic acid, and vitamins at the moment he became paraplegic. Ascending quadriplegia within 2 weeks. |
| Mclean et al. (1994)^25^ | ALL, CNS - | Vincristine, daunorubicin, prednisone, and L-asparaginase. CNS system prophylaxis was started with IT MTX, Ara-C, and hydrocortisone, total 19x IT. | 3m post IT chemo: Bilateral leg weakness, progressed for 3-4 weeks, difficulty walking. 2m later bladder involvement. Babinski present bilateral. Sensory level T10. Vibration and join position were normal. | Dexamethasone. Ascending paraplegia 3 months after IT. Improvement over 5 months and able to walk alone. |
| Mena et al. (1981)^57^ | **1pt:** ALL  **2pt:** ALL, with CNS residual disease. | **1pt:** MOAD IT cytosine arabinoside, and 2400 rads of cranial irradiation. **2pt:** daunorubicin and cytosine arabinoside. IT cytosine arabinoside. Thio-TEPA: IT, radiotherapy (2400 rads). After remission IT Thiothepa. | **1pt:** days later: progressive weakness of the lower extremities, paraplegia. Decreased strength right upper arm. Tendon reflexes and bilateral extensor plantar response absent and sensory loss up to T3-T8. **2pt:** first day after 6e thiotepa: pain in both legs, walking disability. Strength loss, reflexes intact, disturbed sensibility. | **1pt:** Continued treatment. paraplegic within a few weeks after first IT. Died of metastases. **2pt:** several IT doses of hydrocortisone and prednisone. Ascending paraplegia, without improvement. Died of residual disease. |
| Montejo et al. (2019)^76^ | **1pt:** DLBLC, CNS -involvement. **2pt:** ALL, CNS - | **1pt:** Triple IT. **2pt**: dexamethasone, vincristine, MTX, and PEG‐asparagine. MTX + dexamethasone IT. | **1pt:** 10d after third IT chemotherapy: muscle weakness, to paraplegia with urine retention. No deep tendon reflexes, sensory level T1. **2pt:** 8d after last IT chemotherapy: muscle weakness, to paraplegia. | **1pt:** IV methylprednisolone. ascending paraplegia due to lumbosacral polyradiculoneuropathy. No improvement after treatment. **2p:** MTX IT stopped. ascending paraplegia due to lumbosacral polyradiculoneuropathy. No improvement. |
| Murata et al. (2015)^16^ | DBCL, recurrent disease with CNS involvement. | CODOX-M/IVAC (CODOX-M: cyclophosphamide, vincristine, doxorubicin, and high-dose MTX; IVAC: ifosfamide, etoposide, and high-dose cytarabine. | After 5m: dysesthesia bilateral feet. After IT for recurrency (MTX + calcium folinate). 1m later: flaccid paresis of lower limbs, fecal and urinary incontinence. No deep tendon reflexes. | Leucovorin calcium and high dose B12. Ascending paraplegia after 2^nd^ IT. No improvement. Died after 3 months of onset due to disease progression. |
| Ngo et al. (2015)^53^ | T-cell ALL, CNS - | Received six cycles of cyclophosphamide, vincristine, doxorubicin, and dexamethasone (hyper-CVAD) along with prophylactic IT liposomal cytarabine. | 10d after final dose of nelarabine: bilateral lower extremity numbness ascending to mid thoracic region and gait instability, lower extremity weakness, urinary incontinence, and impaired coordination of her bilateral upper extremities. | No treatment. Slightly improved, passed away after reoccurrence of disease. |
| Ostermann et al. (2011)^52^ | 8pt PCNLS | MTX, Ara-C, dexamethasone, vinca-alkaloids, ifosfamide cyclophosphamide. IT liposomal Ara-C. | 7 patients (21%) suffered from an incomplete conus/cauda  syndrome with incontinence for bladder (six) and bowel function (three) or lumbosacral polyradicular paresis (one). Earliest onset after the 2nd IT. The last 5 months after completion of therapy. | Conus/cauda  syndrome with incontinence and bowel function or lumbosacral polyradicular paresis within weeks to months after the first IT. No improvement was noticed after 30 months. |
| Özön et al. (1994)^77^ | Mixed phenotype ALL | Prednisolone, vincristine, daunorubicin, L-asparaginase; triple IT: MTX, Ara-C, prednisolone. | Immediately after IT: Pain back and neck, next day: burning sensation and paresthesia’s lower extremities. Increase neck pain and stiffness. 3d later: leg weakness, urination problems and constipation, deep tendon reflexes diminished. No sensory sensations. Day 20: completely quadriplegic. Progressed to brainstem until dependent of a ventilator. | At day 10: IVIG. Switched to HD methylprednisolone. No benefit. Within 30d complete quadriplegic and dependent on a ventilator. Complete remission, but never gained function back. |
| Park et al. (2013) ^78^ | DLBCL, CNS + | Craniotomy, systemic chemotherapy, with MTX, thiopeta, and vincristine, IT chemotherapy with MTX, Ara-C, and hydrocortisone, intracranial radiation doses. | **After 4 weeks initiation IT:** weakness of the bilateral lower extremities, dysuria, Bilateral hypesthesia to the pain, temperature, vibration, and proprioception below L2 No deep tendon reflexes, and the pathologic reflexes including Babinski sign and ankle clonus were absent. No urination sense and no anal mucosa touch or deep anal pressure showed weakness of the anal sphincter muscle. No bilateral bulbocavernosus reflexes (BCR). | Complete paraplegia after IT (cauda syndrome). No neurological improvements upon discharge and at 14m follow-up visit. |
| Pascual et al. (2008)^79^ | Ph +ALL, q34, 22q11.2. | Induction: vincristine, daunorubicin, cytosine arabinoside, L-asparaginase and prednisone. CNS prophylaxis: IT MTX plus hydrocortisone. | 4d after IT: urinary retention, uncomfortable sensation lower limbs, mild weakness, and unstable gait. Neutropenic fever and bacterial bilateral pneumonia. Progressed to severe flaccid paraparesis and neurogenic bladder dysfunction. Deep tendon reflexes decreased, and sensory level was not found. 2w later: only able to move the right foot without gravity. Deep tendon reflexes absent. Hypopallesthesia from the iliac spine to the foot, proprioception and cutaneous sensations were normal. | IVIG but showed no improvement. Anterior lumbosacral polyradiculopathy with permanent flaccid paraparesis and with urinary incontinence. No improvement after rehab. 15 months after event: died of lung infection. |
| Pinnix et al. (2017) ^17^ | 12pt: ALL | IT MTX or triple IT (MTX, cytarabine and steroids). | All 12 cases: ascending lower extremity paresthesia’s, urinary and/or bowel incontinence and progressive lower extremity weakness. Saddle anesthesia in 7/12pt. All 12pt areflexia and paraplegia. The median number of days after last IT to onset is days 15 (range 3-60 days). 11pt (85%) had additional CNS treatment after the initial appearance of myelopathy because of concern for leukemia infiltration; two of these patients had received radiation therapy, one to the craniospinal axis and the other to T10 through the sacrum. | Repletion with B12 and folate. Two received dextromethorphan, no improvement. Ascending motor weakening to paraplegia, urinary and bowel incontinence, and sensory loss with dorsal column. No improvement after treatment. |
| Pisani et al. (2007)^80^ | ALL in remission. | I.V 6-mercaptopurine, vincristine, MTX and IT MTX, Ara-C and methylprednisolone. | Few hours after the last IT: complaining of weakness lower limbs, sphincter defects. Rapidly tetraplegic, no deep tendon reflexes. No sensory deficit was found. | Dexamethasone treatment without efficacy. Acute motor neuropathy with gadolinium enhancement of anterior roots and cauda. Tetraplegic. No improvement. |
| Resar et al. (1993)^81^ | Burkitt lymphoma. | POG 8617, cyclophosphamide, vincristine, doxorubicin, IT Ara-C, MTX | 20h after 3rd dose IT Ara-C: leg weakness and paresthesia’s. Normal sensation and deep tendon reflexes. 1h later: complete paralysis of the lower extremities, anesthesia below the T10 dermatome, and urinary retention. Within 12h: no spinal cord sensory or motor function, lethargic and confused. Autonomic instability by hypertension and irregular respirations requiring intubation. Ascending brain stem dysfunction. Two days after the onset, mental status improved, and he was able to perceive a sharp touch on his face. | Treatment stopped. High-dose I.V dexamethasone. Ascending myelopathy to brainstem with progressive encephalopathy. Died 10 months after onset of neurological symptoms of recurrent disease. |
| Rolf et al. (2006)^82^ | **1pt:** ALL, CNS relapse. **2pt:** ALL, CNS - | **1pt:** Risk arm of protocol COALL-06-97 including IT MTX during maintenance therapy. **Relapse**: Triple IT **2pt:** maintenance therapy with prophylactic IT MTX three months after the end of intensive chemotherapy. | **1pt:**  During 3rd course within relapse protocol: progressive unilateral hip pain and difficulties in walking, neurogenic bladder, wheelchair bound. Deep tendon reflexes limbs and abdomen absent. Developed muscular weakness of upper extremities, discrete neural autonomous dysregulations, and minor sensitivity deficits. **2pt:** 14d after 2^nd^ IT MTX: high fever, pain in his knees, signs of meningeal irritation. Micturition was increasingly impaired. **1 week later**: progressive weakness of his lower extremities and was diplegic within two weeks. Progressed to upper limbs. | **1pt:** IT continued. Oral substitution of magnesium and high dose vitamin B6: no effects. Immunoglobulin (1 g/kg) two consecutive: No effect. Chemo stopped. Ascending paralysis with urine and bowel involvement. Symptoms remained stable until she died from a bone marrow relapse 7 months later. **2pt:** treated with prednisolone IVIG and HD vitamin B1 and B6. No improvement. 4m later, regained more motoric control of his upper extremities. Remained wheelchair-bound with an atonic paresis of his lower extremities and with minor sensibility deficits while his deep tendon reflexes remained lost. |
| Saito et al. (2013)^30^ | cerebral LBCL. | HD-MTX/Ara-C and repetitive lumbar IT Ara-C and dexamethasone. | 1m after the first round of IT: bilateral numbness in her lower legs. 9m later: gradually developed an unstable gait. Neurological examination revealed a mild lower-limb weakness and a sensory ataxic gait with a positive Romberg’s sign. Pinprick, light touch, vibration sensation, and proprioception impaired below Th10. Deep tendon reflexes normal, Diminished abdominal reflexes and bilateral extensor planter responses. | Delayed ascending paraplegia. Suggested diagnosis of demyelinating myelopathy. Did not improve despite therapy with intravenous administration of vitamin B12 and folic acid. 17m after the first round of chemotherapy, the patient died of recurrent brain lymphoma |
| Saleh et al. (1989)^62^ | Burkitt lymphoma. | Doxorubicin, vincristine, Ara-C, and dexamethasone. IT MTX with cranial irradiation. IT Ara-C. Due to persistence malignancy in CSF: IT Ara-C with hydrocortisone; and MTX. | During 2nd injection Ara-C: shooting pains in the right thigh lasting for 30min. After 3rd day of injection: sudden onset of severe lancing pain in the T10-12 dermatomes radiating to both thighs. LP atraumatic with clear spinal fluid (no malignancy). Within minutes unable to move his legs. Flaccid paralysis both lower extremities with a sensory level ascending to T2. Absent ankle, patellar, and cremasteric reflexes and a negative Beevor’s sign. Sphincter tone diminished. | Reversed Trendelenburg position and received I.V hydration and I.V hydrocortisone. Acute ascending flaccid paralysis (myelopathy) which completely resolved after reversed Trendelenburg position and IV. Hydration and hydrocortisone within 17 hours. Died of systemic disease progression two months later. |
| Santos-Garcia et al. (2008)^35^ | ALL, remission. | IT Ara-C and hydrocortisone as consolidation therapy and I.V vincristine, cyclophosphamide, L-asparaginase, dexamethasone, 6-mercaptopurine and oral MTX. | After 3rd dose of IT over a 3-month period, developed progressive flaccid weakness of lower limbs. Flaccid areflexic paraparesis (2/5), no sensory deficit. | IT stopped and the patient received I.V treatment with methylprednisolone. Acute ascending paralysis consistent of pure motor lumbosacral radiculopathy. Partially recovered within 2m but remained with mild paraparesis. Patient died of pneumonia 3m later. |
| Schwenn et al. (1991)^83^ | 2pt Burkitt Lymphoma | Cytoxan, vincristine, MTX, and Ara-C. Concomitant IT and high-dose IV Ara-C. | Both patients had lower extremity weakness and bowel and bladder dysfunction. 1 after 14d of the 2^nd^ dose if IT. The other 2m after the 2^nd^ course. | First patient recovered fully; 2^nd^ pt stabilized but remained paralyzed. |
| Sherman et al. (2002)^7^ | ALL, pre-B-lineage, aplastic marrow. CSF + | Prophylactic IT MTX, followed by cranial irradiation. Treated with electron beam radiotherapy. An Ommaya reservoir (Heyer-Schulte Corp., Goleta, California) was placed in the CSF space of the frontal convexity, and treatment with IT cytarabine and hydrocortisone. | 1w: progressive loss of sensorimotor function in the lower extremities and urinary incontinence over a 3- to 4-day period. Complete paralysis below the waist. Ascended over a 2-week period to the fourth cervical dermatome. Quadriplegic but did not require mechanical ventilation. | IT therapy was discontinued. Ascending paralysis (myelopathy) into quadriplegia. Remained quadriplegic. He was transferred to a neurorehabilitation unit for ongoing care in died 3 months later. |
| Shintaku et al. (2014) ^21^ | Lymph plasmatic lymphoma | IT MTX and Ara-C. | 7m after beginning of IT: fever, weakness of the lower extremities and unsteady gait. Flaccid paraplegia and urinary and fecal incontinence. 2m later: the spinal cord lesions progressed to the lower thoracic level, and disorientation also appeared and progressed. | Ascending paralysis (myelopathy) 7 months after start IT. Died 4 months after onset of neurological symptoms. |
| Suematsu et al. (2018)^84^ | ALL pre-B. | Vincristine, daunorubicin, L-asparaginase, and prednisolone. Weekly IT MTX, cytarabine, and prednisolone (triple IT) during induction chemotherapy. | After 3rd dose triple IT: incontinence and progressive flaccid paralysis of the legs, weakness of the trunk muscles. Neurological examination revealed areflexia of both legs, with no sensory deficit. | Triple IT stopped and the patient received I.V. methylprednisolone, after which chemotherapy was restarted without triple IT. Ascending paraplegia (lumbosacral polyradiculopathy). After 4 months of rehabilitation, although the strength of her leg muscles gradually improved, they remained unable to bear weight. |
| Tariq et al. (2018)^5^ | ALL, T cell | Hyper-fractionated cyclophosphamide, vincristine, Adriamycin, and dexamethasone (hyper-CVAD. Nelarabine. Haplo-identical bone marrow transplant. As part of the pre- transplant prophylaxis, he was given systemic hyper-CVAD as well as IT MTX. | 1d following the IT infusion of MTX: motor weakness and loss of sensation in bilateral lower extremities into complete paraplegia 6days later. Progressed to complete quadriplegia. | The IT immediately discontinued, high dose systemic and IT steroids. plasmapheresis and IVIG. No improvement. Ascending paraplegia into quadriplegia. Subsequently, the patient developed multiple decubitus ulcers leading to refractory septic shock 5m later. |
| Teh et al. (2007)^14^ | ALL, CNS + | Autologous peripheral blood stem cell transplantation (PBSCT). IT MTX, Ara-C and dexamethasone. | After completion of IT injection (45 min): numbness left lower limb, development of paraplegia and paresthesia of the lower parts of the body. No urinary or bladder incontinence. Sensory loss up to T4. | Symptoms completely resolved 30min after IV hydrocortisone 100 mg and piriton 10 mg. Complete paraplegia within 1 week after IT. Resolved in 30 min day after I.V. hydrocortisone. |
| Thyss et al. (1986)^85^ | ALL, CNS + | Prophylaxis IT MTX and irradiation of the CNS. IT MTX, Ara-C, prednisone. | Transverse myelitis occurred three days after the 8th injection and resulted in permanent paraplegia. | Permanent paraplegia. |
| Von der weid et al. (1991)^58^ | ALL (non-B non-T). | Treatment according to the “SAKK 1976, low risk” protocol, including CNS prophylaxis and 1 course of 1800 R CNS irradiation and IT MTX. | 10 hrs. after TIT: pain in both legs and high- grade fever (39°C). Over 36hr symptoms progressed to a severe myelo-encephalic syndrome with violent headaches, opisthotonos, and walking difficulties. Within 48 hrs. definite paraparesis, there was no Babinski sign and no sphincter disturbances. Complete flaccid paraplegia within 4 days. Preservation of deep and superficial sensation. On the 5^th^ day went into a coma (GS 3). | Paraplegia into coma within 9 days. Passed away by stopping the pulmonary ventilator. |
| Wang et al. (2011)^28^ | ALL | IT MTX, Ara-C, and dexamethasone. | Shortly after IT: complete sensorimotor paraplegia in lower extremities, ascended and affected both upper limbs gradually. Areflexia and flaccid without any sensation. The patient got agitated because of paroxysmal pruritus over the area from both upper extremities to umbilical level (T10). | Placed in reclining position and oxygen delivered as she felt dyspnea. Promethazine 25 mg and dexamethasone 5.0 mg IV. CSF replacement. Diazepam 10 mg was intravenously injected (for general muscles jerks during CSF replacement). Acute paraplegia into quadriplegia. Three days after the CSF replacement, the patient could walk freely in the ward. A 3-month follow-up revealed that she recovered uneventful. |
| Watterson et al. (1994)^86^ | **1pt:** Burkitt  **2pt:** Burkitt  **3pt:** Burkitt  **4pt:** Burkitt  **5pt:** Burkitt  **6pt:** Burkitt  **7pt:** AML/ALL biphenotypic  **8pt:** CNS lymphoma  **9pt**: Burkitt | **1pt:** IT MTX, Ara-C, hydrocortisone, and HD MTX, Thiotepa  **2pt:** IT MTX, Ara-C and hydrocortisone and CRT  **3pt:** IT MTX, Ara-C  **4pt:** IT MTX, Ara-C and hydrocortisone and CRT  **5pt:** IT MTX, Ara-C and hydrocortisone and CRT  **6pt:** IT MTX, Ara-C and hydrocortisone  **7pt:** IT MTX, Ara-C  **8pt:** IT MTX, Ara-C and spinal radiation  **9pt**: IT MTX, Ara-C and spinal radiation | All presented with ascending paraplegia. Symptoms specified below:  **1pt:** Onset: 7d, cord destroyed below T6.  **2pt:** Onset: 29d, ascending to brain.  **3pt:** Onset: 42d, paraparesis.  **4pt:** Onset: 42d, paraplegia,  **5pt:** Onset: 8w, paraplegia,  **6pt:** Onset: 11w, paraparesis,  **7pt:** Onset: 11w, quadriplegia,  **8pt:** Onset: 12w, paraplegia,  **9pt**: Onset: 13w, paraplegia, | **1pt:** Partial recovery  **2pt:** Death due tox  **3pt:** Partial recovery  **4pt:** Partial recovery  **5pt:** Slowly recovering.  **6pt:** Partial recovery  **7pt:** Partial recovery (DOD)  **8pt:** Partial recovery (DOD)  **9pt**: recovery |
| Yi et al. (2015)^18^ | Burkitt. | Cyclophosphamide, vincristine, and prednisolone, doxorubicin, and MTX, etoposide with HD cytarabine. | Between the 10th and 11th round IT”:  difficulty urinating, gait ataxia and bilateral lower extremity weakness. Proprioception and sense of light touch decreased. Cerebellar function tests normal. 9d after the symptom onset, the 11th IT triple therapy was administered. Day 27: bilateral facial palsy and decreased facial sensation developed. Progressed to upper extremities. | Copper (II) sulphate pentahydrate 4 mL was given over 15 minutes I.V based on the suspicion that reduced copper level might have induced his neurologic condition. Ascending paraplegia due to myelopathy. Eventually died of respiratory failure. |
| **Re-run** |  |  |  |  |
| Bidikian et al. (2021) ^8^ | T-ALL, CNS + | Hyper-CVAD, IT MTX and cytarabine. Maintained on POMP. For recurrence: BFM protocol (vincristine, prednisone, l-asparaginase, daunorubicin) + triple IT chemotherapy. | Day 15 BMF: tingling sensation and weakness of lower extremities bilaterally, ascended over three days to hips. Decreased motor power (3/5), absent deep tendon reflexes and absent Babinski bilaterally. The rest of neurological exam, including the sensory exam, was normal. Urinary retention, sensory deficit of light-touch, pinprick, and vibration sensation up to the T10 dermatome. | Gabapentin 300 mg three times per day. Folate replenishment. IV methylprednisolone 1 mg/Kg daily. 10 mg/Kg along with IV leucovorin 20 mg four times per day and intramuscular cyanocobalamin 100mcg daily. Symptoms progressed. Patient passed away from septic shock. |
| Cheong et al. (2021) ^87^ | T-ALL. | Cyclophosphamide, vincristine, doxorubicin, and dexamethasone. IT MTX and Ara-C injections for CNS prophylaxis. | 3d after 6th IT: sudden onset of bilateral lower limbs weakness. Neurological examinations revealed flaccid paraparesis with muscle power of ⅗. 2w later: worsening bilateral lower limbs' weakness and urinary retention. Muscle power of the lower limbs was ⅖ with bilateral extensor plantar reflexes. | IVIG for GBS. 5-day course of IV methylprednisolone was given, followed by oral prednisolone. 2w after IVIG, progressed. Her condition gradually improved with rehabilitation. At 6-months follow up, she was able to ambulate with assistance and her lower limbs power improved to ⅘. |
| Chiu et al. (2021)^11^ | AML, CNS + | Induction idarubicin and cytarabine (I3A7); bone marrow and CSF positive -> IT MTX, haploidentical bone marrow transplantation, followed by haploidentical peripheral blood stem cell transplantation (complete remission). | 6w after last IT MTX:  ascending weakness of the lower extremities; urinary incontinence and severe low back pain. Decreased pain and proprioception below L4 level and absence below S3. Deep tendon reflexes hyperactive over bilateral lower extremities, accompanied with bilateral ankle clonus. Muscle power of bilateral proximal and distal legs was 2 and 1 on a scale of 1 to 5, respectively. | Leucovorin, prednisone and vitamins B12. Physical therapy was also arranged. Before discharge, she was able to walk over 100 m without device assistance. |
| Landolfi et al. (2023)^88^ | ALL | LAL1913 protocol; CNS prophylaxis IT MTX and dexamethasone. | 23d after first IT: lower limb weakness. Neurological examination showed flaccid paraplegia: hip flexion (MRC 1/5 on the left, 2/5 on the right). Deep tendon reflexes absent. No sensory, sphincter, and/or autonomic disturbances were detected. MRC sum score for lower limbs was 3/30. | IVIG 0.4 g/kg/day for 5 consecutive days, followed by high-dose IV methylprednisolone 1 g/day for 5 consecutive days and intensive physical rehabilitation. Three months after symptom onset, the patient was able to walk with bilateral support, with steppage. |
| Rodriguez et al. (2022) ^10^ | B-ALL, expressing CD19, CD10 and CD81. CSN + | Cyclophosphamide, daunorubicin, vincristine and prednisone (CALGB regimen). IT MTX, IV cyclophosphamide, mercaptopurine, cytarabine, vincristine and L-asparaginase. MADIT and fludarabine, idarubicin, G-CSF and cytarabine systemic chemotherapy. | 20d after MADIT. Bilateral numbness and sensory abnormalities. in the legs (to umbilicus), gait impairment, urinary incontinence. Distal weakness of the legs; deep tendon reflexes absent in ankles and plantar responses extensor. Impaired proprioception, pain and temperature sensation below Th-6. Tabetic gait and Romberg sign was present. | Interrupted IT chemotherapy and gave folate. After a few days, there was progressive improvement in proprioception, gait and distal leg weakness. Two months later, he could walk unassisted.  However, his condition progressed, with massive brain oedema with CNS infiltration and death. |

MADIT IT regimen (MTX, arabinoside and dexamethasone), G-CSF: granulocyte colony-stimulating factor, POMP”: 6-mercaptopurine, vincristine, methotrexate and prednisone, MOAD: (MTX, vincristine, doxorubicin, and dexamethasone), R-Mega CHOP: rituximab (R) into cyclophosphamide, doxorubicin, vincristine, and prednisone), R-ICE: Rituximab, ifosfamide, carboplatin, etoposide, R-FMD: Rituximab, fast mimicking diet, Hyper-CVAD: cyclophosphamide, vincristine sulphate, Adriamycin, dexamethasone. CALBG: Cancer and Leukemia Group B, R-DHAP: Rituximab, dexamethasone, cytarabine, cisplatin.

**Table S3**: detailed description of inclusions and exclusion.

| **Rf** | **Population** | **Initial therapy disease** | **Initial presentation after therapy** | **Diagnostic tools event** | **Treatment after adverse event** | **Conclusion** |
| --- | --- | --- | --- | --- | --- | --- |
|  | | | | | | |
| **1** | Male, 68y, DLBCL, no CNS involvement, C-MYC positive. | R-CHOP (rituximab, cyclophosphamide, doxorubicin, vincristine, prednisolone). R-DHAP (rituximab, dexamethasone, Ara-C, cisplatin) for progressive disease. Triple IT Ara-C (15 mg MTX, 10 mg dexamethasone and 40 mg  Ara-C). | 2d after triple IT: nausea and vomiting, 2d later rapidly ascending, irreversible sensory, motor and autonomic dysfunction and paraplegic. | MRI spinal cord revealed a central hyperintense signal in T2 weighted sequences interpreted as  myelitis, from the medullary cone up to the T9. CSF revealed protein elevation, no malignant cells. | HD methylprednisolone: no improvement. Systemic chemotherapy was continued with one cycle of rituximab, ifosfamide, carboplatin and etoposide (R-ICE) but no Ara-C. | Paraplegic within 4 days after IT and HD Ara-C I.V. No improvements after treatment. Remained stable. |
| **2** | Female, 29y, T-ALL, no CNS involvement. | PETHEMA LAL-AR but failed. FLAG-IDA achieving MRD. Going for Allo-HSCT). Rescue therapy: nelarabine  was started (1500 mg/m2 IV on days +1, +3, and +5 every 21 days) together with prophylactic triple IT therapy. | During the first days of nelarabine treatment: headaches and self-limited paraesthesia’s in feet and fingertips. On day +30 of nelarabine (day +3 of clofarabine and cytarabine treatment): complained of dysesthesias in lower limbs, progressively ascending during the following days and leading to significant paraplegia with paresis of upper limbs in addition to dysautonomia (urine retention and constipation). | First days: CSF negative for malignant cells. 30 + days: (MRI) showed a weak signal hyperintensity on T2-enhanced images in dorsal portion of the cervical and thoracic spinal cord. Repeated MRI (day 13) showed progressive disease. | Vitamin B6 and B12 high dose, dexamethasone 4mg/12h for days. 4 times plasma exchange. No improvement. | Paraplegic after triple IT and nelarabine. No improvement after treatment. Remained stable. |
| **Rf** | **Population** | **Initial therapy disease** | **Initial presentation after therapy** | **Diagnostic tools event** | **Treatment after adverse event** | **Conclusion** |
| **3** | Female, 3y, ALL, no CNS involvement. | IT MTX, Ara-C, and hydrocortisone, I.V. vincristine, L-asparaginase, cyclophosphamide, oral MTX, and 6-mercaptopurine. | 12 days after starting treatment: progressive lower extremity flaccid weakness, inability to walk, and mild upper extremity weakness over a 2-week period, absent patellar and ankle tendon reflexes, and normal sensation. | Serum creatinine-kinase, TSH, calcium, electrolytes, ESR, complete blood count, blood cultures normal. CSF: 0 white  blood cells/mm3, absence of malignant cells, elevated concentration (107 mg/dl), normal glucose level (55 mg/dl), and normal electrophoresis. Campylobacter Jejuni, Borrelia burgdorferi,  mycoplasma, human immunodeficiency viruses 1 and 2, cytomegalovirus, GM1, asialo-GM1, GD1B, GQ1B antibody titre normal. MRI gadolinium: enhancement ventral roots. NCS normal, F wave absent. Needle EMG showed absent muscle activation. | Treated with 1.7 g/kg of IVIG, divided into two doses given on 2 consecutive days, for a possible acute immune demyelinating polyradiculopathy, and within 3 days proximal leg strength improved. | Progressive ventral polyradiculopathy after IT, treated with IVIG. Improvement after 4 months and able to walk again. |
| **4** | Female, 24y, ALL-L1 (pre-B), no CNS involvement | Hyper-CVAD (cyclophosphamide/Adriamycin/vincristine/dexamethasone/HD MTX/Ara-C). IT MTX (12 mg). | Two days after IT MTX: unable to urinate for nine hours. At the same time, she became constipated for six days. | MRI with gadolinium: enhancement in the cauda equina region, CSF free of leukemic cells. | IT stopped. | Acute urinary retention and constipation after IT. After 21 days regained complete function of bladder and bowel. |
| **5** | Male, <7y, ALL. | St. Jude Total XI and XIII protocols.  IT MTX. | Within 3 days after IT: difficulty in urination and defecation and developed quadriplegia into coma. | Contrast enhancement of fibres of cauda equina. | N.A. | No improvement over time. Died 2.5 years after onset. |
| **Rf** | **Population** | **Initial therapy disease** | **Initial presentation after therapy** | **Diagnostic tools event** | **Treatment after adverse event** | **Conclusion** |
| **6** | Female, 10y, B-ALL standard risk, no CNS involvement. | IT and/or HD MTX (5 g/m2), Cytarabine, cyclophosphamide, mesna,  ondansetron, thioguanine, trimethoprim, sulfamethoxazole. | 11 days after IT: bilateral limb weakness, inability to walk. | N.A. | N.A. | Myelopathy after IT. |
| **7** | Male, 9y, Burkitt lymphoma. | Induction chemotherapy IT (total 10 doses) MTX (12 mg/dose); cytosine  arabinoside (70 mg/dose); and hydrocortisone (50 mg/dose); and  I.V. vincristine, cyclophosphamide, mesna, Adriamycin (doxorubicin), and methylprednisolone. Consolidation with I.V. VP-16 (etoposide) and ifosfamide. 1800 cGy cranial to C3. | 24h after last IT (within 5.5 weeks): paraesthesia’s and progressive numbness and weakness of both lower extremities. 2d later unable to walk. Physical examination revealed an ascending paraparesis, with intact muscle strength and sensation in the upper extremities and 2/5 muscle strength in the lower extremities. Diffuse numbness to the nipple level and incontinence of urine. By day 79: complete quadriplegic. | MRI thoracic and lumbar: normal. Repeated MRI up to 49 days: (T1- and  T2-weighted) and gadolinium-enhanced images demonstrated decreased signal within a markedly widened cervical cord to the level  of T4. EMG: diffuse polyradiculopathy. | Dexamethasone. No improvement. | Diffuse polyradiculopathy into quadriplegia. No improvement after steroids. Died of respiratory failure. |
| **8** | Male, 17y, AML FAB M4Eo, | BFM 1998 Interim protocol but had to receive repeated (17) IT injections with cytarabine, prednisolone, and TT and also (24 Gy). | Toxic cauda equina syndrome with incontinence and permanent paraplegia. | N.A. | N.A. | Toxic cauda equina syndrome with incontinence and permanent paraplegia. |
| **9** | Male, 65, AML, no CSF involvement. | VAPA chemotherapy (vincristine 2 mg I.V. on days 1 and 5; Adriamycin 10 mg/m2 on days 1, 3 and 5; prednisone 40 mg/m2 orally, daily for 5 days; and cytosine arabinoside 80 mg/m' per day as a continuous infusion for 5 days). CNS prophylaxis: 6x IT Ara-C (total 84 mg). Concurrent whole brain irradiation (2550 rads). | 2w following the last dose of IT: unsteadiness and instability of gait, weakness of his lower extremities and urinary incontinence not associated with headache, visual disturbances or paraesthesia’s. Positive findings on neurologic examination, revealed a broad-based gait with normal cerebellar function, diffuse weakness of the legs both proximally and distally, marked position sense loss, sense loss to the hips, diminished touch and position sense distally, a positive Romberg, hyperactive knee jerks, absent ankle jerks and bilateral extensor plantars. Upper normal. | CSF: protein of 50 mg/100 ml, glucose 68 mg/100 ml, 1 mononuclear cell, 3 RBC’s, and was culture negative.  Other determinations included a nonreactive Hinton, vitamin B-12 715 pg/ml, (normal 150-700 pg/ml), and folate 8.4 ng/ml (normal 3-11 ng/ml). | N.A. | Paraplegia within 2 weeks following the last dose IT. Marked improvement over time (3 months) and able to walk. Died of GI bleeding. |
| **Rf** | **Population** | **Initial therapy disease** | **Initial presentation after therapy** | **Diagnostic tools event** | **Treatment after adverse event** | **Conclusion** |
| **10** | Female, 61y, DLBCL, no CNS involvement. | Salvage therapy with rituximab, dexamethasone, high-dose cytarabine, and cisplatin (R-DHAP). Previously completed 6 cycles of rituximab, cyclophosphamide, Adriamycin, vincristine, and prednisone (R-CHOP) with 12mg IT MTX prophylactically in each cycle. | 1d after first IT MTX 12 mg with cytarabine 50 mg: non-positional headache, photophobia, nausea without vomiting, and double vision, rectal incontinence, and urinary retention. Weakness progressed to bilateral lower extremity paralysis. Diminished reflexes in the bilateral patellar and Achilles tendons, positive Babinski on the right, and diminished sensation to light touch | 6d after symptom onset repeat LP again yielded negative cerebrospinal fluid (CSF) for malignant cells, total protein elevated to 131 mg/dL, glucose and total cell count were within normal limits, and gram stain and culture were negative. MRIs revealed mild enhancement of the ventral and dorsal nerve roots of the cauda equina, particularly at T12-L3, and diffuse central spinal cord signal abnormality most prominent from T6-L2. Brain (MRI) showed symmetric FLAIR signal abnormality in the brainstem and cerebellum without diffusion restriction or abnormal enhancement. | N. A | Polyradiculitis, no recovery, progressed soon and passed away due to systemic lymphoma. |
| **Rf** | **Population** | **Initial therapy disease** | **Initial presentation after therapy** | **Diagnostic tools event** | **Treatment after adverse event** | **Conclusion** |
| **11** | **1pt:** 70y, B-ALL Ph-  **2pt:** 59y, B-ALL Ph-  **3pt:** 18y, Burkitt, CNS relapse  **4pt:** 32y, B-ALL Ph-, CNS relapse  **5pt:** 48y, B-ALL, relapse  **6pt:** 50y, CML Ph+, CNS relapse  **7pt:** 35y, B-ALL Ph-, CNS relapse | **1pt:** Hyper-CVAD, rituximab, inotuzamab, ozogamycin.  **2pt:** Hyper-CVAD, Ofatumumab  **3pt:** Rituximab, hyper-CVAD, anti CD19 Ab: MOR0028.  **4pt:** Hyper-CVAD, evrolimus, clofarabine, idarubicin, cytarabine,  rituximab, vincristine, anti CD19  Ab. 92 cycles: MOR0028.  **5pt:** CALGB, CD19 Ab Maytansine conjugate, Hyper-CVAD, inotuzamab ozogamycin.  **6pt:** Imatinib, Allogenic stem cell with cyclophosphamide.  **7pt:** Hyper-CVAD, POMP maintenance,  Augmented hyper-CVAD. | **1pt: 2 days after IT MTX:** Back pain, urinary/faecal  incontinence, paraplegia, LE  areflexia, saddle anaesthesia,  absent Babinski’s.  **2pt: 1 month after IT MTX:** Urinary/faecal incontinence,  saddle anaesthesia, T6 sensory  level, paraplegia, LE areflexia,  absent Babinski’s.  **3pt: 2 months after IT:** T8 sensory level, paraplegia, LE  areflexia, bilateral positive Babinski’s,  **4pt: 7 months after IT:** Saddle anaesthesia, paraplegia, urinary/faecal incontinence, LE areflexia,  Bilateral positive Babinski’s.  **5pt: 1 week after IT:** Saddle anaesthesia, T8 sensory level, LE paraplegia, urinary/focal  incontinence, absent LE reflexes,  absent Babinski’s.  **6pt: 2 months after IT:** Urinary/faecal incontinence, paraplegia, T5 sensory level, absent LE reflexes, and positive Babinski.  **7pt: 2 months after IT:** Urinary/faecal incontinence, saddle anaesthesia, paraparesis, bilateral positive Babinski’s. | **MRI**: 5/7 had abnormalities in the dorsal columns. The other 2 had cauda enhancement and negative for CSF for cancers cells. CSF elevated protein in  five patients. All seven eventually developed elevated protein.  (MBP), was assessed in two patients and was  significantly elevated in both. Cytological  normal in all seven patients. | **1pt:** Dexamethasone,  dextromethorphan, IVIG 5 days.  **2pt:** Dexamethasone, IVIG x 5 days, Vitamin B12.  **3pt:** Dexamethasone, Vit B12, IVIG 95 days.  **4pt:** Dexamethasone, folic acid,  CSRT.  **5pt:** IVIG x 5 days.  **6pt:** CSRT.  **7pt:** Pregabalin, dextromethorphan. | **1pt:** Neurological improvement but  died 1 month organ failure.  **2pt:** No neurological improvement.  Alive at 6 months post onset symptoms.  **3pt:** No neurological  improvement. Died  5 weeks after onset from organ failure.  **4pt:** No neurological  improvement. Died  2 months post onset.  **5pt:** No neurological improvement. Died  2 months post onset.  **6pt:** No neurological improvement 6 months post onset. Died  7 months post onset.  **7pt:** Neurological improvement post rehab but developed LMD 2 months later with neurological deterioration. |
| **12** | Male, 21y, ALL. | Five chemotherapy sessions with I.V doxorubicin and vincristine, and IT MTX, ARA-C and dexamethasone. | During the fifth IT: developed dizziness and headache, and after the administration had finished, he lost consciousness. On regaining  consciousness he was completely deaf, paraplegic with abolished sensory modalities up to his chest  and unable to control sphincters. | **MRI**: normal. Negative for HIV and syphilis. **CSF**: absent cell count, normal protein, glucose and lactate. **SEP** showed impairment in spinal cord. | Prednisone (1 mg/kg) was prescribed for 21 days,  without improvement. | At follow-up 1 month later, strength had improved to 3/5, but patient was unable to weight bare and sphincter function was unchanged. |
| **Rf** | **Population** | **Initial therapy disease** | **Initial presentation after therapy** | **Diagnostic tools event** | **Treatment after adverse event** | **Conclusion** |
| **13** | Female, 12y, ALL. | Cyclophosphamide, vincristine (oncovin), cytosine arabinoside and oral prednisolone. IT cytosine arabinoside. | After 4^th^ intra-thecal therapy, she began to complain of urine and faecal retention. After 2 months: restarted and he complained of weakness and tingling sensation in the lower limbs with flaccid paraplegia. Sensation normal. | T2-weighted gradient recalled echo images confirmed the signal changes in the spinal cord. | After first event: Further chemotherapy was withdrawn for 2 months. Again, chemo stopped. | She became quadriplegic by of the month, she had generalized convulsions with laboured breathing and died a few days later. |
| **14** | 1pt, ALL, paediatric | BFM 86 protocol | Quadriparesis within 14 days after IT. | CSF and MRI negative. | N.A | Recovered within 72 hours. |
| **15** | 2/290 pt, haematological malignancy. | IT MTX 15 mg and dexamethasone 4 mg. For patients with BL, triple  therapy with MTX 15 mg, Ara-C 40 mg and dexamethasone 4 mg is used during the entire study period according to the German BFM Regimen schedule. | N. A | N. A | N. A | N. A |
| **16** | Male, 73y, blastic plasmacytoid dendritic cell neoplasm, relapse with CNS metastases, ALL. | 5 doses IT cytarabine initial treatment. 7 doses IT cytarabine (12mg), MTX (50mg) and hydrocortisone (50mg) with relapse. | 1 year after chemotherapy: Progressive weakness and paraesthesia of the lower extremities, and disturbed vibration and proprioception. Bladder and bowel dysfunction. | **MRI**: high signal intensity in bilateral posterior columns in the cervicothoracic spine.  **Lab:** negative lab for metabolic or infectious causes including homocysteine levels. | Folic acid and cyanocobalamin. | Quadriplegic passed away. PA: biopsy of the lower thoracic spine dorsal column revealed cord destruction and diffuse macrophage infiltration. No tumour infiltrate or microorganism. |
| **Rf** | **Population** | **Initial therapy disease** | **Initial presentation after therapy** | **Diagnostic tools event** | **Treatment after adverse event** | **Conclusion** |
| **17** | 1395 paediatric patient’s B/T ALL, age (1.9-15y), (n=15/17 spinal neuropathy, 2 loss to follow up), FRALLE 93 study. | Triple IT therapy (TIT) methylprednisolone (MP), MTX and Cytarabine adjusted for age.  One case occurred during induction, two during consolidation, seven during intensification and five during maintenance therapy.  The median TIT number was 10 (2–18), and onset occurred a median of 13 days after the last injection. | **Spinal involvement** **n=7**; 2/7pt flaccid quadriplegia, sensory and sphincter disorders. 5/7 patients solely motor involvement; paraplegia in three cases with pyramidal signs (Babinski sign, brisk osteo-tendinous reflexes).  **Anterior horn involvement n=3:** Rapidly progressive purely motor neuropathy (flaccid paraplegia).  **Peripheral involvement n=5:** Sensory-motor-radiculo-neuritis, two during consolidation, seven during intensification and five during maintenance therapy. The median TIT number was 10 (2–18), and onset occurred a median of 13 days after the last injection. | **Spinal involvement** **4/7** **pt**: Spinal MRI abnormal with peri-spinal contrast enhancement in three cases and epidural enhancement in two cases. **Anterior horn involvement:** 3pt lesions on nerve conduction study and electromyography (EMG). MRI normal,  **Peripheral involvement:** Elevated proteinorachia (2/5); one child had CSF hypercellularity; EMG and NCS showed axonal lesions. MRI normal. | ALL treatment was modified in 11/15 cases: TIT stopped in 9 cases and replaced by CNS irradiation in two cases and by HD MTX in one case. Cytarabine was stopped in one case and vincristine in 8/15 cases. For 3pt vincristine toxicities were difficult to rule out because of the transient and moderate nature of the disorders. | 6/15 children fully recovered. 9/15 had chronic sequels and recovered very slowly. Spinal involvement had the worst functional prognosis. 8/9 children showed persistent paraplegia and need for urinary drainage. |
| **18** | **1pt:** Female, 14y and **2pt:** female, 16y, AML, No CNS involvement. | Protocol #8101. Total IT doses of MTX 90 mg, hydrocortisone 138 mg, and Ara-C 180 mg, all of which were administered in the lumbar region. Cranial RTX (12x200rad). No spinal radiation. | **Pt 1:** 4 months after IT chemotherapy, the patient developed hypesthesia and weakness of the lower extremities + occasional urinary and faecal incontinence.  **Pt 2**: 2-weeks left knee pain, 4 days of left leg weakness and right foot numbness, and early morning urinary retention. Within 1 month, neck pain, increasing left knee pain, worsening left hemiparesis, and frequent urinary incontinence. | **Pt 1**: CSF normal, CSF cytology normal, CT normal, myelogram normal. EEG and EMG normal.  **Pt 2:** CSF normal (myelin basic protein less than 4 mg/dl). CT normal. Myelogram normal. | **Pt1/Pt2:** Dexamethasone was administered without appreciable symptomatic improvement.  Chemotherapy was discontinued. | **Pt1**: Medulla and cervical region. Multifocal gelatinous softening, loss of gray-white demarcation. Microscopy: regional demyelination, axonal degeneration, gliosis, and spongiosis. neuronal loss, gliosis, and oedema.  **Pt 2:** cervical oedema after 2 months of the first onset of symptoms. Symptoms progressed, and she passed away. No autopsy. |
| **19** | Male, 29y, ALL + CNS involvement | Doxorubicin, vincristine, cytosine arabinoside, and dexamethasone, IT MTX 24 mg per dose (total 8 injections). 2500 rad cranium. | After 8^th^ injection, paraplegia and atonic bladder. | Myelogram and lumbosacral spine films were normal. CSF clear. | Folic acid 3 mg intravenously and dexamethasone 2 mg with no improvement in neurologic function. | 6 weeks after onset: return of deep tendon reflexes, complete left leg function and partially right leg function. |
| **Rf** | **Population** | **Initial therapy disease** | **Initial presentation after therapy** | **Diagnostic tools event** | **Treatment after adverse event** | **Conclusion** |
| **20** | **1pt:** male, 67, DLBCL  **2pt:** female, 74y, MALT  **3pt:** male, 57y, DLBCL  **4pt:** female, 57, DLBCL | **1pt:** 4 (R-MegaCHOP93, R-CHOP91), IT courses before event: 4  **2pt:** 6 (R-FMD), IT courses before event: 6,  **3pt:** 11 (R-CHOP98, R-ICE93), IT courses before event: 3  **4pt:** 2 (R-Mega CHOP), IT courses before event: 2. | **1pt:** 24d after starts: Conus medullaris/cauda equina syndrome.  **2pt:** 24 days after start: Conus medullaris/cauda equina syndrome.  **3pt:** 24 days after start: Conus medullaris/cauda equina syndrome.  **4pt:** 6 days after start: Conus medullaris/cauda equina syndrome. | MRI in all normal. CSF: first patient not tested; second patient, elevated protein; third patient, increased protein and mild pleocytosis; fourth patient, normal. | **1pt:** none.  **2pt:** 7 days 20/mg dexamethasone.  **3pt:** none.  **4pt:** IT withdrawn. | **1pt:** complete resolution.  **2pt:** slight improvement.  **3pt:** slight improvement.  **4pt:** complete resolution. |
| **21** | Female, 6y, ALL, no CNS involvement. | Vincristine, daunorubicin, prednisone, L-asparaginase, and two doses of triple IT chemotherapy, achieving complete remission. LLA-VII-9 1 therapy protocol. | 12h after the last lumbar puncture, she presented with a severe headache, backache, opisthotonos, and fever. 10 days later: paraparesis, urinary incontinence, and diplopia. | MTX levels in CSF negative. Normal CSF biochemistry.  Negative serology for CMV, VZV, EBV, HSV1 EBV, HIV, T. Gondi.  Elevated MBP  EEG: slow activity.  MRI: negative. | High-dose intravenous steroids, intravenous immunoglobulin, and folic acid were administered without clinical response. | Flaccid quadriplegia with respiratory failure and bulbar involvement (facial diplegia, dysphagia, diplopia with normal ocular motility). T1-MRI: spinal atrophy after 6 months. |
| **22** | Male, 16y, Burkitt lymphoma | I.V chemotherapy: cyclophosphamide 400 mg (d 1-4), vincristine 2 mg (d 5), MTX 6g (d 5), ifosfamide 1.6 g (d 5-9), prednisolone 120 mg (d 1-4). IT cytosine arabinoside (Ara-C 40 mg), MTX (MTX, 15 mg) and dexamethasone (4 mg). | Severe central neuropathy within 24 h after the first IT injection. 8 h post IT, weakness and paraesthesia of both legs combined with a urinary retention. After 3 d he had vomiting, dizziness and disorientation. Symptoms progressed tongue weakness, diminished facial sensations and ascending paraesthesia up to dermatome thoracal 2. Became complete quadriplegic. | CSF: normal, no leukemic cells.  MRI: negative.  After >10d: cerebrospinal fluid leucocytes to 30 μL, erythrocytes 2 μL, no tumour cells. Protein concentration 1.2 g/l), albumin and immunoglobulins were slightly increased, lactate was 5.6 mmol/l. | Stopped IT. | After 10d, complete quadriplegia and dependent on respiratory ventilator. patient passed away without recurrence of the tumour. |
| **23** | Female, 42y, ALL, no CNS involvement. | Hyper-CVAD with IT MTX and cytarabine. | Five weeks after her **first dose** of IT MTX (12 mg), she developed numbness in both lower limbs. Lower limb weakness, areflexic paraparesis, impaired vibration sense in her toes and a positive Romberg’s sign.  **Second dose days later:** no function in the lower limbs, sensory level at T10 and bilateral extensor plantar responses. | **After first IT MTX dose**: MRI normal. Normal nerve conduction, CSF no malignant cells, cytoalbuminic dissociation (white blood cells = 0; protein=1.03).  **After second dose**:  T2-weighted hyperintensity dorsal columns, T4 to conus medullaris, no contrast enhancement. CSF normal. Folic acid, B12 (1043 pmol/L), thyroid, full blood, sediment, liver, kidney all normal.  ANA, dsDNA, m-protein negative. | **First dose:** She received intravenous (IV) immunoglobulin for presumptive Guillain–Barré syndrome.  **After second event:** folic acid supplements. She also received five days of IV methylprednisolone. No effects. | Complete paraplegia with bladder dysfunction, and late MRI changes in the dorsal and lateral columns. Patient stayed disabled and passed away due to intracranial haemorrhage. |
| **Rf** | **Population** | **Initial therapy disease** | **Initial presentation after therapy** | **Diagnostic tools event** | **Treatment after adverse event** | **Conclusion** |
| **24** | Male, 16, ALL, CNS involvement, but in complete remission. | Purin-Ethol dally and MTX (MTX) I.V. weekly. IT MTX. | Six weeks after 7^th^ IT MTX treatment, Patient developed paraplegia, sensory level at T2 which ascended to the C7 in 2 weeks. Descended again and reached T8 where it remained. | N. A | N. A | Paraplegic and sensory levels at T8. Passed away 5 months after the onset of neurological symptoms. Postmortem: brain normal. Cone shaped necrotizing spinal cord segment T8-T10. Spared white matter. Minimal lymphocyte infiltrate. |
| **25** | 2 adults, ALL. | Cyclophosphamide, daunorubicin, vincristine, and prednisolone, cytarabine, leucovorin. IT MTX + hydrocortisone. | Second round IT. | N. A | Stopped IT, no improvement. | Myelopathy after IT MTX. No improvement. |
| **26** | **1pt**: Male, 18y, c-ALL.  **2pt:** Female, 52y, AML. 1x it cytarabine. | It liposomal cytarabine 50mg + 4mg oral dexamethasone. GMALL 7/03. | **1pt:** 4 weeks after the last IT dose. (Total 9 doses).  **2pt:** 7 days after first IT dose. | **1pt:** MRI negative.  **2pt:** MRI negative. CSF: signs of meningitis but viral and bacterial was negative. | 1pt: N.A.  2pt: dexamethasone. | **1pt:** cauda equina syndrome. Remission.  **2pt**: paraplegia but resolved after dexamethasone and progressive disease. |
| **27** | Female, 42y, pre-B-ALL. | Induction hyper-CVAD, completing all 6 doses of liposomal cytarabine. | Developed slow onset of faecal incontinence, urinary hesitancy, and perineal numbness, progressing after cycle 4 of MTX and cytarabine. | LP and MRI were negative. 2 months later: MRI showed enhanced sacral roots. | Corticosteroids. | Mild improvement. |
| **28** | Female, 31y, lymphoblastic lymphoma | Daunorubicin 30 mg, cyclophosphamide 750 mg, vincristine 2 mg and prednisone 60 mg. IT cytarabine (ARA-C), MTX and prednisolone, doxorubicin (14.5 mg). | Hypoesthesia, paraparesis and incontinence progressively developed over 7 days. | CSF: Protein of 1.58 g/l.  CSF after 10 days: inflammation of the meninges and the spinal cord, protein of 1.95 g/dl (3.74 after 18 days), white cell 23/ml (7% polymorphonuclear, 32% lymphocytes and 61%monocytes). MRI severe arachnoiditis of the conus medullaris and cauda equina. | Accidently doxorubicin IT: CSF exchange at a rate of 20 cm^3^/h, total of 500 cm^3^. IV methylprednisolone (500 mg/day) 7 days and immunoglobulin (22 g/day) for 5 days without improvement. | Hypoesthesia, paraparesis, incontinent. Treatment didn’t improve symptoms. Arachnoiditis and myelopathy. |
| **Rf** | **Population** | **Initial therapy disease** | **Initial presentation after therapy** | **Diagnostic tools event** | **Treatment after adverse event** | **Conclusion** |
| **29** | Female, 42y, T-ALL. **Relapse:** mediastinal and bone marrow relapse, CSF positive. | Hyper-CVAD + 8 cycles IT MTX and cytarabine. **Relapse:** Nelarabine and 2 IT cycles MTX + cytarabine + Hydrocortisone. | **Relapse:** 24 h after last dose, ascending bilateral limb numbness, progressed but stopped at the lower cervical region. | **MRI** **first week**: negative.  EMG: negative. CSF, serum and B12: negative.  **MRI 6th week**: abnormal high  T2 and STIR signal posterior cord, lower cervical segments to entire thoracolumbar to conus medullaris. Gadolinium enhancement T11 to conus. DSEP normal cortical response T2 bilaterally, absent cortical responses with stimulation T4 and T6 bilaterally. Normal angiography spine. | No intervention. Additional 2 IT cycles MTX and total body radiation. 7 weeks after event 5d high dose methyl prednisone without effect. | Complete dorsal myelopathy and loss of proprioception. No worsening of symptoms. Dependent on a walker. Passed away 11 months after event. |
| **30** | 2/120 pt, 48y average, lymphoma. 1pt: CNS involvement, 2pt: No CNS involvement. | IT MTX and cytarabine. | Both cases: CTCAE grade 1 myelopathy/ cauda equina syndrome,  saddle paraesthesia involving S4–S5 dermatomes, without  incontinence and motor dysfunctions. | N.A. | No treatment. | It resolved without  any dedicated treatment after 2 and 3 days. |
| **31** | 4/47 pt, 3pt cauda equina syndrome, 1pt sacral nerve injury. | IT triple therapy (MTX, cytosine arabinoside, and hydrocortisone) s injected according institutional protocol (ALL-BFM 95). | Urinary retention, faecal  incontinence, and lower extremity weakness. | N.A. | N.A. | Cauda equina syndrome resolved within months, sacral nerve injury was permanent. |
| **32** | Male, 22y, NHL High grade, T-cell, immunoblastic | 100 mg IT Ara-C, IV doxorubicin (40 mg/2), cisplatin (100 mg/m2), and  Ara-C (1.5 g/m2). | 8 h after induction, the patient had hypoxia, widened arterial-alveolar gradient, tachycardia. Thirty h after induction, blurred vision progressed to total blindness  and paraplegia, sensory level T4. weakness of both arms, followed by coma and unreactive dilated  pupils. Flaccid tone in all extremities Deep tendon reflexes absent in arms and legs after 48h. | **MRI**: minimal swelling, no lesions. **CSF** 1d after event: protein level 480 mg/dl, glucose of 63 mg/dl. Cytologic no lymphomatous involvement. EMG: motor absent, sensory intact. **MRI later:** extensive necrosis medulla. | N.A. | Ascending quadriplegia up to the medulla oblongata. Extensive transverse coagulative necrosis with neither inflammatory nor macrophage reaction. |
| **Rf** | **Population** | **Initial therapy disease** | **Initial presentation after therapy** | **Diagnostic tools event** | **Treatment after adverse event** | **Conclusion** |
| **33** | **1pt**: male, 5y, ALL, CNS prophylaxis,  **2pt**: male, 4y, ALL, CNS Prophylaxis.  **3pt**: male, 22m, ALL, CNS prophylaxis. | **Systemic**: 6-mercaptopurine, vincristine, dexamethasone in 1pt and 2pt, l-asparaginase, vincristine, prednisone in 3pt. **IT: 1pt:** IT MTX. 3 times within 3 months (3x 12 mg).  **2pt**: IT MTX, 11 times (132 mg in 10 months). **3pt:** IT MTX, 6x in 3 months (48 mg total). | **1pt:** bilateral leg weakness 2 days after 3rd IT MTX, progressive, unable to walk, reflexes and sensory normal. Incontinence.  **2pt**: frequent falls after 11^th^ IT MTX, areflexia and muscle weakness, sensory normal.  **3pt:** leg weakness after last IT MTX, unable to walk. Areflexia. | **1pt**: CSF: protein (56 mg/dL), IgG (9,36 mg/24h), no oligoclonal bands or elevated MBP. CSF cytology normal.  EMG and (NCS) axonal neuropathy, active denervation Lumbar spine MRI: gadolinium enhancement anterior roots of the cauda. **2pt**: CSF normal, no oligoclonal bands or MBP. EMG and NCS axonal neuropathy, no denervation. Gadolinium enhancement of anterior roots of cauda equina was demonstrated. **3pt:** CSF elevated protein (131 mg/dL) and IgG (17,3 mg/24h). positive oligoclonal bands. Cytology and MBP normal. Peroneal axonal neuropathy, no active denervation on EMG and NCS. Spinal MRI: gadolinium enhancement of the anterior roots at the cauda equina. | **1pt:** 3 days methyl prednisone 30mg/kg iv.  **2pt**: 3 days methyl prednisone 30mg/kg iv.  **3pt:** 3 days methyl prednisone 30mg/kg iv. | **1pt:** complete recovery after 2 months.  **2pt:** recovered quickly after treatment.  **3pt:** mild improvement, able to stand, but legs remained spastic. |
| **34** | Male, 38y, Ph+ ALL. No CNS involvement. 3yrs later CNS relapse, testicular swelling.  1y later another CNS relapse. 2^nd^ relapse. And resistant disease. | UKALL IX protocol into successful remission. Maintenance 6-mecarptopurine, IT MTX for prophylaxis. 3y later CNS relapse treated with UKALL IX and radiation. First relapse: IT MTX and Ara-C (total 10). Second relapse: IT MTX and Ara-C + hydrocortisone. Resistant disease: IT Mitoxantrone 3 times. | After second IT mitoxantrone (2mg), local pain. Third dose (1mg), again local pain. 3-4 days later: severe pain in legs, paraplegia within 2 weeks. Absent leg reflexes, no sensory loss or alteration in bowel or  bladder function. | The CSF blast count fell to less than 1/ microlite after. | N.A. | Complete paraplegic within 2 weeks of mitoxantrone without sensory problems, bowel or bladder involvement. Stayed in the chronic phase. |
| **Rf** | **Population** | **Initial therapy disease** | **Initial presentation after therapy** | **Diagnostic tools event** | **Treatment after adverse event** | **Conclusion** |
| **35** | Male, 24y, T-LBL | CVAD (cytarabine, etoposide, MTX, L-Asparaginase, doxorubicin, vincristine with total administered doses: cytarabine 53 g/m2 and MTX 6.8 g/m2). 5 times prophylaxis triple therapy. Oral 6-mercaptopurine. Nelarabine I.V. | After the fourth nelarabine gift: ascending sensory problems starting in the legs to the anterior abdominal wall, muscle weakness within 10 days. After IVIG, continued to ascend to upper limbs. | Diagnostic lumbar puncture: negative for CNS involvement, mild increase in CSF protein content (67 mg/dL). PCR Epstein-Barr virus and cytomegalovirus as well as bacterial culture of the CSF all negative. MRIs of the brain and the spine negative. EMG suggestive for Guillain-Barré–like syndrome of acute polyradiculopathy. **MRI day 31**: still negative. **Day 56:** MRI: pathological signal intensity changes T3 and extending throughout the spinal cord, no signs of expansion, a characteristic of myelopathy of the spinal cord. | **Day 10**: IVIG 2g/kg. **Day 33**: IV high dose steroids. **Day 40**: vitamin supplementation and plasma exchange. | Complete paraplegia, bilateral plantar extensor reflex, urinary and bowel dysfunction. Hypoesthesia stabilized at C5-C6. Regained complete function after a year of rehab therapy. |
| **36** | Male, 53y, T-ALL, no CNS involvement. | 8x (TIT, consisting of in  cytarabine, Ara-C, 50 mg + MTX, MTX, 12.5 mg + dexamethasone, DEX, 4 mg)  CNS relapse (4x liposomal cytarabine 50mg. TIT restarted prophylaxes, 6x total. | 4 days after the last TIT (14 x + Ara-C): Incontinence, rapidly progressing ataxic gait, and lower limb.  hypoesthesia below Th10 level and complete paraplegia up to T4 within 10 days. | Spine MRI hyperintensity on T2 weighted images involving the dorsal  columns from Th12 to Th8, up to cervical region within 15 days. CSF: negative for disease. CSF 2 weeks later: neutrophilic  pleocytosis and hyperproteinorrachia, no residual disease, aquaporin, MOG and paraneoplastic antibodies negative. | B12 and folic acid. Plasma exchange and steroids. | Complete paraplegia with urinary and bowel involvement, with MRI changes up to the cervical tract. No improvement after therapy. |
| **Rf** | **Population** | **Initial therapy disease** | **Initial presentation after therapy** | **Diagnostic tools event** | **Treatment after adverse event** | **Conclusion** |
| **37** | 5/75 had an event. Burkitt or L3 ALL. | Patients received an initial 5-day course cyclophosphamide and  Prednisone. All received CNS prophylaxis chemotherapy and irradiation. Twelve doses IT chemotherapy. Cranial irradiation (24 Gy in 12 fractions) after cycle 3 chemotherapy. For treatment details See reference. | Transverse myelitis developed between cycles four and six in four  patients, but one patient developed transverse myelitis as a  late complication approximately 5 months after treatment  was completed. | N.A. | N.A. | Transverse myelitis after IT developed +- 4 cycles, and 1 pt 5 months after the last cycle. |
| **38** | Female, 25y, B-ALL, no CNS involvement. | VPDL regimen of 2 mg IV vincristine, 60 mg/m2 prednisolone, 45 mg/m2  intravenous daunorubicin, 4,000 units/m2 intramuscular L-asparaginase. On day 42, complete remission. Day 43: IT MTX 12mg, 40mg cytarabine, 50mg hydrocortisone. | Day 47: ascending leg weakness, unable to walk. No tendon reflexes, mild sensory deficit. Urinary retention. | CSF: first LP blasts but follow up LPs did not show any blasts. EMG  and NCV studies consistent with myelopathy and neuropathy in the motor and left median  axons. MRI lumbar spine, diffuse gadolinium enhancement of the anterior roots (ventral roots). | 3 days 30mg/kg methyl prednisone. | Irreversible paraplegia: Myelopathy or ventral neuropathy with urinary retention. Eight months after the first MRI scan, a follow-up T-2 weighted MRI revealed heterogeneous high intensity lesions in the thoracic spinal cord, in addition to severe atrophy. |
| **39** | Female, 44y, T-cell lymphoma, CNS involvement. | HCVAD/MA, IT MTX and cytarabine. Second line treatment: gemcitabine and vinorelbine Third line treatment: ICE (ifosphamide, carboplatin, etoposid Switched to pralatrexate. During the second event: 2 doses of IT MTX 12 mg and 9 doses of IT cytarabine 100 mg over an 18-week period. | **First event:** After 3rd pralatrexate: bilateral flaccid lower limb paralysis  (Power 0/5), bilateral upper limb weakness (power 3/5), areflexia, a  T7/8 sensory level, and bilateral loss of dorsal column function in her feet.  Progressive tetraplegia. **Second event:** (after restarting treatment) re-presented with bilateral flaccid lower limb paralysis (power 0/5), bilateral upper limb weakness (power 3/5), areflexia, a T7/8 sensory level, and bilateral loss of dorsal column function in her feet. | **First event:** MRI spine revealed extensive epidural hematoma extending from the skull base to the lumbar spine, causing cord compression at level T4. to T7. **Second even:** MRI spine revealed new dorsal column signal change from C2 throughout the spine to the conus, no signal of cord compression. Lab: B12. folic acid and copper normal. CSF: clean form residual disease. | **First event:** conservative treatment resolved the problem. | Ascending paraplegia into tetraplegia. Passed away within two weeks of the events. |
| **Rf** | **Population** | **Initial therapy disease** | **Initial presentation after therapy** | **Diagnostic tools event** | **Treatment after adverse event** | **Conclusion** |
| **40** | **1pt:** female, 28y, T-LBL, no CNS involvement.  **2pt**: female, 60y, FL CNS involvement. | **1pt:** 8x MTX 12 mg, Ara-C 30 mg, and hydrocortisone 24 mg.  **2pt:** 9x i.t. MTX 12 mg, Ara-C 30 mg, and hydrocortisone 50mg. | **1pt**: weakness of bilateral lower extremities, paraesthesia, and voiding difficulty gradually developed. Vibration and joint position senses of both legs were severely impaired. Urinary and stool incontinence developed. **2pt:** bilateral weakness in the lower leg. Sensory impairment in the loves-stock pattern, and severe impairment of the vibration and joint position senses of both legs. Urinary incontinence developed later. | **1pt:** MRI: symmetrical hyperintensity at the posterior columns of the spinal cord, lower cervical cord down to the conus medullaris on T2-weighted images. **2pt:** Symmetric hyperintensity at the posterior columns of the lower cervical cord down to the conus medullaris on T2 WI without abnormal enhancement. **Both:** No enhanced contrast images. B12 and folic acid were normal. | **1pt:** B12 and folic supplements. | Ascending paraplegia without improvement. MRI changes were almost identical. Both passed away, but not as a consequence of the event. |
| **41** | Male, 37y, CNS DLBCL. | Rituximab, MTX, procarbazine, and vincristine; triple IT therapy MTX, cytarabine, and hydrocortisone. | 1 week after last cycle of IT: tingling and numbness of both lower limbs into quadriplegia. Difficulty walking, unsteady gait, and a girdle sensation around lower chest bilaterally. Impaired vibration and joint position sensations in lower limbs with sluggish deep tendon reflexes and extensor planters. | **First MRI**: MRI spine and nerve conduction studies normal. **Second MRI:** (2 weeks later): showed a hyperintense signal in the posterior aspect without contrast enhancement. | Injection methyl cobalamin, folic acid, and vitamins at the moment he became paraplegic. | Ascending quadriplegia within 2 weeks. |
| **42** | Female, 13y, ALL, no CNS involvement. | Vincristine, daunorubicin, prednisone, and L-asparaginase. CNS system prophylaxis was started with IT MTX (190m), Ara-C (950mg), and hydrocortisone (950mg), total 19x IT. | 3 months post IT chemo. Bilateral leg weakness progressed during 3-4 weeks, not walk without support. 2 months later bladder involvement. Babinski present bilateral. Sensory level T10. Vibration and join position were normal. | **CSF:** no blasts, mild protein increase. Bacterial, fungi or TB: negative. Viral culture polio virus, echovirus, coxsackievirus, HSV, and mumps virus negative.  **MRI**: T1 weighted decreased signal central portion of cervical and upper dorsal spinal cord. The post gadopentetate dimeglumine images show enhancement in lateral column. | 4 mg dexamethasone three times a day for 5 days followed by a decreasing dose over 2 weeks. | Ascending paraplegia 3 months after IT. Improvement over 5 months and able to walk alone. |
| **Rf** | **Population** | **Initial therapy disease** | **Initial presentation after therapy** | **Diagnostic tools event** | **Treatment after adverse event** | **Conclusion** |
| **43** | **1pt:** Male, 33y, ALL  **2pt:** Female, 32, ALL, with CNS residual disease. | **1pt:** MOAD (MTX, vincristine, doxorubicin, and dexamethasone), IT cytosine arabinoside, and 2400 rads of cranial irradiation. **2pt:** daunorubicin and cytosine arabinoside. IT cytosine arabinoside. Thio-TEPA: IT 10 mg for four days and radiotherapy (2400 rads). After remission 2x IT Thiothepa. | **1pt:** days later, he developed progressive weakness of the lower extremities, became paraplegic. Decreased strength right upper arm, a weakness upper and lower extremities. Tendon reflexes and bilateral extensor plantar response absent and sensory loss up to T3-T8. **2pt:** first day after 6e thiotepa: pain in both legs, walking disability. Strength loss, reflexes intact, disturbed sensibility. | **1pt:** CSF clean. **2pt**: CSF: no malignant cells. Protein: 126 mg/dl, glucose 62 mg/dl  Myelogram normal,  EMG showed evidence of bilateral denervation potentials in L4-S I nerve roots. | **1pt:** Continued treatment. **2pt:** several IT doses of hydrocortisone and prednisone. | **1pt:** paraplegic within a few weeks after first IT. Died of metastases.  **2pt:** ascending paraplegia, without improvement. Died of residual disease. **Histopathologic**: changes in the spinal cord localized to white matter and consisting of sponginess, loss of myelin, axonal swelling, fibrillary gliosis, and presence of macrophages. Polymorphonuclear leukocytes, mononuclear cells, and fibrinoid necrosis or vascular occlusion were not seen in any of the patients. |
| **44** | **1pt:** Male, 58y, DLBLC, no CNS involvement. **2pt:** Female, 26y, ALL. | **1pt:** Triple it. 3x. Total dose IT MTX 36mg. **2pt**: dexamethasone, vincristine, MTX, and PEG‐asparagine. Five doses of MTX + dexamethasone IT (total dose of 60 mg) over a course of 5 months. | **1pt:** 10 days after third IT chemotherapy: muscle weakness, to paraplegia with urine retention. No deep tendon reflexes, sensory level T1. **2pt:** 8 days after last IT chemotherapy: muscle weakness, to paraplegia with urine retention. No deep tendon reflexes. | **1pt:** CSF parameters within normal limits.  NCS and EMG 1 week after onset showed absence of F wave in both lower limbs with minimal amplitude decrease and normal latency in CMAP. MRI: no gadolinium enhancement. **2pt:** CSF: elevated protein (96.4 mg/dL), normal cells.  NCS and EMG studies at 10 days and follow-up (22 and 90 days). MRI demonstrated gadolinium enhancement of the anterior roots of the cauda equina. | **1pt:** IV methylprednisolone. **2p:** MTX IT stopped. | **1pt:** ascending paraplegia due to lumbosacral polyradiculoneuropathy. No improvement after treatment. **2pt:** ascending paraplegia due to lumbosacral polyradiculoneuropathy. No improvement. |
| **Rf** | **Population** | **Initial therapy disease** | **Initial presentation after therapy** | **Diagnostic tools event** | **Treatment after adverse event** | **Conclusion** |
| **45** | Male, 59, DBCL, recurrent disease with CNS involvement. | CODOX-M/IVAC (CODOX-M: cyclophosphamide, vincristine, doxorubicin, and high-dose MTX; IVAC: ifosfamide, etoposide, and high-dose cytarabine. | After 5 months of therapy: dysesthesia of his bilateral feet. After recurrency of therapy: 2x IT MTX (15mg) with the addition of calcium folinate. 1 month later: flaccid paresis of lower limbs, faecal and urinary incontinence. No deep tendon reflexes. | Serum B12 l2788pg/mL, all other tests normal. (Copper, 132μg/dL; total homocysteine, 8.7nmol/mL; and folic acid, 7.6ng/dL).  **CSF**: cytoalbuminic dissociation (mononuclear cells, 1/mm3; protein, 123mg/dL),  glucose level 47mg/dL (blood sugar, 98mg/dL). MBP 3087pg/mL and CSF homocysteine 1.2nmol/mL, Negative  CSF oligoclonal bands, soluble interleukin-2  receptor, CSF cytology, and polymerase chain reaction testing for various viruses.  **MRI** revealed signal hyperintensity on T2-weighted imaging  in the posterior funiculus of his cervical spinal cord and in the lateral and posterior funiculi of his thoracic spinal cord. | Leucovorin calcium at 60mg/day and high dose B12. | Ascending paraplegia after 2^nd^ IT. No improvement. Died after 3 months of onset due to disease progression. |
| **46** | Female, 37y, T-cell ALL. | She received six cycles of cyclophosphamide, vincristine, doxorubicin, and dexamethasone (hyper-CVAD) along with four courses of prophylactic IT liposomal cytarabine. | 10d after final dose of nelarabine: bilateral lower extremity numbness. numbness ascended to her mid thoracic region, and she developed gait instability, lower extremity weakness, urinary incontinence, and impaired coordination of her bilateral upper extremities. | **MRI:** 5 weeks later increased T2 signal in dorsal columns. | N.A. | Slightly improved, passed away after reoccurrence of disease. |
| **Rf** | **Population** | **Initial therapy disease** | **Initial presentation after therapy** | **Diagnostic tools event** | **Treatment after adverse event** | **Conclusion** |
| **47** | 8/33 PCNLS, 65 years, range 32–76 years. | MTX, Ara-C, dexamethasone, vinca-alkaloids, ifosfamide cyclophosphamide. IT liposomal Ara-C. | Seven patients (21%) suffered from an incomplete conus/cauda  syndrome with incontinence for bladder (six) and bowel function (three) or lumbosacral polyradicular paresis (one). Earliest onset after the second IT. The last 5 months after completion of therapy. | **CSF** analysis was performed in six patients. No increased cell count, slightly increased total protein of up to 66 mg/dl in four cases. **MRI:** scans of the lumbosacral spine were negative for leptomeningeal infiltration or arachnoiditis. | N.A. | Conus/cauda  syndrome with incontinence and bowel function or lumbosacral polyradicular paresis within weeks to months after the first IT. No improvement was noticed after 30 months. |
| **48** | Male, 14y, mixed phenotype ALL. | Prednisolone, vincristine, daunorubicin, L-asparaginase; triple IT: MTX, Ara-C, prednisolone. | Immediately after IT: Pain back and neck, next day: burning sensation and paraesthesia’s lower extremities. Increase neck pain and stiffness. 3 days later: leg weakness, urination problems and constipation, deep tendon reflexes diminished. No sensory sensations. Day 20: completely quadriplegic. It progressed to brainstem until dependent of a ventilator. | **MRI**: thickening cauda equina. (arachnoiditis). Spinal cord looked normal. Gadolinium shows thickened cauda roots and thickened pia around distal conus medullaris. **CSF**: protein elevated 283 mg/dl, glucose 59 mg/dl, no leukemic blast cells. Normal CT. **EMG**: extensive motor denervation lower limb. Follow up: ascending myelopathy. | At day 10: gamma globulin 400mg/kg iv. 6 days. Switched to high dose methylprednisolone. 30mg/kg 5 days. No benefit. | Within 30 days complete quadriplegic and dependent on a ventilator. Complete remission, but never gained function back. |
| **49** | Male, 58y, DLBCL, | Craniotomy, systemic chemotherapy, with MTX, thiopeta, and vincristine, IT chemotherapy 9 times, with MTX 15 mg, Ara-C 40 mg, and hydrocortisone 50, intracranial radiation 30 Gy doses. | **After 4 weeks initiation IT:** weakness of the bilateral lower extremities, dysuria, Bilateral hypesthesia to the pain, temperature, vibration, and proprioception below L2 level bilateral No deep tendon reflexes, and the pathologic reflexes including Babinski sign and ankle clonus were absent. No urination sense and no anal mucosa touch or deep anal pressure showed weakness of the anal sphincter muscle. No bilateral bulbocavernosus reflexes (BCR). | **CSF**: normal opening pressure, protein level 78.1 mg/dL, glucose more than 70% of blood sugar. No white blood cells nor malignant cells. Decreased (CMAP) amplitude of the right limb. No CMAP response left EDB muscle. Delayed F-wave latencies. H-reflex was absent in the bilateral tibial nerves. Delayed cortical motor evoked potentials. Bilateral somatosensory cortices no responses after tibial stimulation. **EMG** revealed abnormal spontaneous activities. Areflexic neurogenic bladder. Cauda equina syndrome. **CT**: no lesions. no neurological improvements upon discharge and at 14 months follow-up visit. | N.A. | Complete paraplegia after IT (cauda syndrome). No neurological improvements upon discharge and at 14 months follow-up visit. |
| **50** | Male, 30y, Ph +ALL, q34, 22q11.2. | Induction: vincristine, daunorubicin, cytosine arabinoside, L-asparaginase and prednisone. CNS prophylaxis: IT MTX plus hydrocortisone. | Four days after IT: urinary retention, uncomfortable sensation in his lower limbs, mild weakness and unstable gait. Neutropenic fever and bacterial bilateral pneumonia. Leg weakness progressed to severe flaccid paraparesis and neurogenic bladder dysfunction. Deep tendon reflexes decreased, and sensory level was not found. Two week later: only able to move the right foot without gravity. Deep tendon reflexes absent. Hypopallesthesia from the iliac spine to the foot, proprioception and cutaneous sensations were normal. | **CSF**: elevated protein (163 mg/ dL), normal glucose level (65 mg/dL), mononuclear pleocytosis (white blood count, 21 cells/mm3), increased immunoglobulin synthesis (15.6 mg/%). **Serologic**: Mycoplasma, Campylobacter Jejuni, Borrelia burgdorferi, Enterovirus, cytomegalovirus, herpes simplex virus, herpes zoster virus, varicella-zoster virus, liver viruses and human immunodeficiency viruses 1 and 2 were negative. **Antibodies:** GM1, GB 1B, GQ 1B not detected. Absence of neoplastic cells. **MRI with gadolinium:** normal. reduced amplitude in muscle action potentials with no alteration in velocity patterns. Sensory normal. No F waves. Reduced motor unit potential recruitment. | I.V immunoglobulin but showed no improvement. | Anterior lumbosacral polyradiculopathy with permanent flaccid paraparesis and with urinary incontinence. No improvement after rehab. 15 months after event: died of lung infection. |
| **51** | 12pt, ALL. | IT MTX or triple IT (MTX, cytarabine and steroids). Median number of IT treatments was 17 (range 3–36). | All 12 cases, ascending lower extremity paraesthesia’s, urinary and/or bowel incontinence and progressive lower extremity weakness. Saddle anaesthesia in 7/12pt. All 12pt were noted to have areflexia and paraplegia. The median number of days after last IT to onset is days 15 (range 3-60 days). 11pt (85%) had additional CNS treatment after the initial appearance of myelopathy because of concern for leukaemia infiltration; two of these patients had received radiation therapy, one to the craniospinal axis and the other to T10 through the sacrum. | Initial **MRI**: normal in 7/13pt (54%). Re-review of these scans showed abnormalities revealed subtle T2 changes had been present in 4 of those cases. **CSF:** no evidence of leukaemia in any pt, 12/12 elevated protein, MBP elevated all 4/4 tested. Low serum folate levels 6/8 tested pt (62%). Serum vitamin B12 levels 9/10 tested pt normal. homocysteine levels elevated 4/4pt tested. Levels of methylmalonic acid, were normal, suggesting a deficiency in folate, but not in vitamin B12, in these patients. | Repletion with B12 and folate, no improvement. Two received dextromethorphan, no improvement. | Ascending motor weakening to paraplegia, urinary and bowel incontinence, and sensory loss with dorsal column. No improvement after treatment. |
| **52** | Female, 4y, ALL in remission. | I.V 6-mercaptopurine, vincristine, MTX and IT MTX, Ara-C and methylprednisolone. 5d doses of IT over one year (total dose of MTX 60 mg). | Few h after the last IT: complaining of weakness lower limbs, sphincter defects. Rapidly tetraplegic, no deep tendon reflexes. No sensory deficit was found. | **CSF:** elevated protein level without oligoclonal bands or elevation of MBP; cytology normal. **NCS** complete absence of median, ulnar and posterior tibial motor responses, preserved velocity and amplitude of compound muscle action potentials of the facial nerve. Median, ulnar and sural sensory velocities were also normal with sensory action potentials mildly depressed (median 12.7 µV, ulnar 4.2 µV, sural 10.3 µV). **EMG**: no motor unit potentials or signs of active denervation. **MRI:** gadolinium enhancement anterior roots of the cauda equina, anterior horns and anterior spinal nerve roots. | The patient was given dexamethasone (2 mg/kg/day for one month) without efficacy. | Acute motor neuropathy with gadolinium enhancement of anterior roots and cauda. Tetraplegic. No improvement. |
| **53** | Male, 16y, Burkitt lymphoma. | POG 8617, cyclophosphamide, vincristine, doxorubicin, IT Ara-C, MTX. | 20 h after 3rd dose IT Ara-C: leg weakness and paraesthesia’s. Normal sensation and deep tendon reflexes. One hour later: complete paralysis of the lower extremities, anaesthesia below the T10 dermatome, and urinary retention. Within 12 h: no spinal cord sensory or motor function, lethargic and confused. Autonomic instability by hypertension and irregular respirations requiring intubation. Ascending brain stem dysfunction. Two days after the onset, mental status improved, and he was able to perceive a sharp touch on his face. | **Serum:** electrolyte and glucose tests normal results, no evidence of tumour lysis syndrome.\. **MRI** **3 h after onset**: swelling of the thoracic and lumbar spinal cord. **Non contrast CT:** normal. Normal opening pressure. **CSF**: contained 197 leukocytes/uL and 102 erythrocytes/ uL, protein was 1390 mg/dl and glucose 61 mg/dl. MBP elevated at 28 ng/dl. 24h after IT Ara-C, concentration Ara-C was 2.1 pM in the lumbar CSF and 1.17 pM in the ventricular CSF. These values decreased after 48h. **EMG:** extensive motor neuron injury upper cervical to the lower lumbar regions., **MRI**: focal abnormalities in the cerebellum and brain stem, swelling of the entire spinal cord with thickening of the nerve roots. | Treatment stopped. High-dose I.V dexamethasone (100 mg for one dose, then 24 mg intravenously every 6 hours), Hyperventilation and mannitol therapy were begun to prevent increased intracranial pressure, and the patient was treated with phenytoin to prevent seizures. | Ascending myelopathy to brainstem with progressive encephalopathy. Died 10 months after onset of neurological symptoms of recurrent disease. |
| **Rf** | **Population** | **Initial therapy disease** | **Initial presentation after therapy** | **Diagnostic tools event** | **Treatment after adverse event** | **Conclusion** |
| **54** | Adults with Burkitt leukaemia or lymphoma | Detailed description is given in the article. **Cohort 1**: 2 IT per cycle.  **Cohort 2:** 1 IT per cycle. | N.A. | N.A. | N.A. | **Cohort 1:** 3/58 pt developed transverse myelitis after IT. **Cohort 2:** showed no transverse myelitis, indicating the lower frequency of IT can prevent an event. |
| **55** | **1pt:** Female, 14y, ALL, CNS negative. 17 months later CNS relapse. **2pt:** Male, 7y, ALL, CNS negative. | **1pt:** Risk arm of protocol COALL-06-97 including three IT applications of MTX during maintenance therapy. **Relapse**: IT injections (MTX 12 mg, Ara-C 30 mg, Pred 10 mg).  **2pt:** maintenance therapy with prophylactic IT MTX three months after the end of intensive chemotherapy. | **1pt:** During 3rd course within relapse protocol: progressive unilateral hip pain and difficulties in walking. **10d** after last IT chemotherapy (total of 5 triple drug IT), acute retention of urine due to neurogenic bladder. Increasing weakness of both legs and wheelchair bound. No muscle strength in thighs, marked weakness of trunk muscles, weakly dorsal flex big toes. Deep tendon reflexes limbs and abdomen absent. Developed muscular weakness of her upper extremities, discrete neural autonomous dysregulations and minor sensitivity deficits. **2pt:** 14 days later several hours after 2^nd^ IT MTX: high fever, pain in his knees, signs of meningeal irritation. Micturition was increasingly impaired. **1 week later**: progressive weakness of his lower extremities and was diplegic within two weeks. Deep tendon reflexes of lower extremities lost, no pathological reflexes. Trunk and upper extremities affected resulting in a complete paresis of his upper arms and a marked weakness of his forearms. decreased. Respiration was normal at rest with a decreased capacity to cough and laugh. | **MRI:** negative. CSF**,** EEG, ECG and abdominal ultrasound: normal. Laboratory: low serum levels of protein, albumin, IgA and IgM, elevated liver enzymes and bone marrow aplasia with 0.7 × 109/l leukocytes and relative monocytosis. White cell count below 2 × 109/l. **Viral infection:** negative by serology and PCR analysis from blood, stool and CSF. **Auto inflammatory** was negative. **Serum inflammation** markers: negative. **EMG and NCS** velocity confirmed neurogenic disease and a severe mixed axonal-demyelinating. **CSF** later: low monocyte count and elevated levels of protein, albumin, IgA, IgM and IgG as a sign of a dysfunctional blood-brain-barrier. MBP (8.5 ng/ml, N: 0–2). **2pt:** CSF normal. MRIs, EEG, fundus examination and viral serology showed normal. Negative inflammation markers. Neurophysiological examinations showed axonal lesion of motoric nerves, initially in the lower and later in the upper extremities. | **1pt:** IT continued. Oral substitution of magnesium and high dose vitamin B6: no effects. Immunoglobulin (1 g/kg) two consecutive: No effect. Chemo stopped. **2pt:** treated with prednisolone (2 mg/kg/d) for one month until there was no further progression of symptoms. Two cycles of immunoglobin G (1 g/kg/d, days 1–4) repeated after two weeks, and HD vitamin B1 and B6 therapy did not improve his symptoms. Four months later, he regained more motoric control of his upper extremities. Making continuously slow progress, he was also able to urinate more frequently on his own. | **1pt:** ascending paralysis with urine and bowel involvement. Symptoms remained stable until she died from a bone marrow relapse 7 months later. **2pt:** regained more motoric control of his upper extremities. Eventually able to urinate on his own. Remained wheelchair-bound with an atonic paresis of his lower extremities, minor sensibility deficits and his deep tendon reflexes remained lost. |
| **Rf** | **Population** | **Initial therapy disease** | **Initial presentation after therapy** | **Diagnostic tools event** | **Treatment after adverse event** | **Conclusion** |
| **56** | Female, 35y, cerebral LBCL. | 3 courses of HD-MTX/Ara-C and repetitive lumbar IT Ara-C (100 mg) and dexamethasone (4 mg). | One month after the first round of IT: bilateral numbness in her lower legs despite. Nine months later: gradually developed an unstable gait. Neurological examination revealed a mild lower-limb weakness and a sensory ataxic gait with a positive Romberg’s sign. Pinprick, light touch, vibration sensation, and proprioception were impaired below the level of the Th10. Deep tendon reflexes normal in all limbs but diminished abdominal reflexes and bilateral extensor planter responses. | **MRI after first month:** normal. Macrocytic anaemia, reduced thrombocyte count. Routine biochemistry, autoantibodies, and thyroid function test normal. Serum vitamin B12 level in the lower limits of normal [286 pg/ml (normal range 180–914)], and serum folate and copper were slightly reduced. **CSF:** white blood cell count of 1/μl, a protein level of 24 mg/dl, and increased MBP; 502 pg/ml). NCV and compound muscle action potentials normal, SEP demonstrated a normal, but prolonged P 40 responses (44.1 ms). **MRI after 9 months:** T2-WI MRI signal intensity in the bilateral and dorsal columns in the thoracic cord, extending from Th3 to Th10. | **After first event:** She received a repetitive course of IT Ara-C (40 mg) and prednisolone (4 mg) with or without IT MTX (15 mg) for 6 months, along with whole-brain radiotherapy for 1 month. Did not improve despite therapy with intravenous administration of vitamin B12 and folic acid. Seventeen months after the first round of chemotherapy, the patient died of recurrent brain lymphoma. | Delayed ascending paraplegia. Suggested diagnosis of demyelinating myelopathy. Seventeen months later patient died of recurrent lymphoma. |
| **Rf** | **Population** | **Initial therapy disease** | **Initial presentation after therapy** | **Diagnostic tools event** | **Treatment after adverse event** | **Conclusion** |
| **57** | Male, 54y, Burkitt lymphoma. | Doxorubicin, vincristine, Ara-C, and dexamethasone. IT MTX (12.5 mg/dose) and hydrocortisone (15 mg/dose) with cranial irradiation (3,000 cGy total dose). IT Ara-C (30 mg). Due to persistence of malignant cells in the spinal fluid, IT Ara-C (45 mg/m2) with hydrocortisone (30 mg) on Days 1, 2, and 3; and MTX (12.5 mg) on Day 4. | During 2nd injection of Ara-C, transient shooting pains in the right thigh lasting for 30 minutes. After day 3 injection: sudden onset of severe lancing pain in the T10-12 dermatomes radiating to both thighs. LP atraumatic with clear spinal fluid. Within minutes unable to move his legs. Flaccid paralysis of both lower extremities with a sensory level at T10 eventually ascending to T2. Absent ankle, patellar, and cremasteric reflexes and a negative Beevor’s sign. The toes did not move to plantar stimulation and the sphincter tone was diminished. consistent with transverse myelopathy. | EMG no abnormalities.  No MRI. | Reversed Trendelenburg position and received I.V hydration and I.V hydrocortisone. In this position, the patient’s sensory level descended, and 17 hours from the time of onset, his myelopathy resolved completely without any residual sequela. He was subsequently discharged but died of systemic disease progression two months later. | Acute ascending flaccid paralysis (myelopathy) which completely resolved after reversed Trendelenburg position and IV. Hydration and hydrocortisone within 17 hours. Died of systemic disease progression two months later. |
| **58** | Male, 45, ALL, remission. | IT Ara-C and hydrocortisone as consolidation therapy and I.V vincristine, cyclophosphamide, L-asparaginase, dexamethasone, 6-mercaptopurine and oral MTX. | After third dose of IT over a 3-month period, developed progressive flaccid weakness of lower limbs. Flaccid areflexic paraparesis (2/5), no sensory deficit. | Haematological, serologic and CSF all normal. NCS and EMG showed data consistent with motor lumbosacral polyradiculopathy and marked acute denervation involving L2-S2 roots bilaterally. Gadolinium enhancement of cauda equina anterior roots was observed on MRI. 2 months later: MRI normal, CSF still clean from malignant cells. | IT was stopped and the patient received I.V treatment with methylprednisolone (1000 mg/day for 5d). | Acute ascending paralysis consistent of pure motor lumbosacral radiculopathy. Partially recovered within 2 months but remained with mild paraparesis. Patient died of pneumonia 3 months later. |
| **59** | 2/5 pt, paediatric (3 and 15y, Burkitt. | Cytoxan, vincristine, MTX, and Ara-C. Concomitant IT and high-dose IV Ara-C. | Both patients had lower extremity weakness and bowel and bladder dysfunction. 1 after 14 days of the 2^nd^ dose if IT. The other 2 months after the 2^nd^ course. | EMG and CT: normal.  CSF: 1 normal protein, 1 224 while receiving WBC transfusion for polymicrobial sepsis. | N.A. | First patient recovered fully; 2^nd^ pt stabilized but remained paralyzed. |
| **Rf** | **Population** | **Initial therapy disease** | **Initial presentation after therapy** | **Diagnostic tools event** | **Treatment after adverse event** | **Conclusion** |
| **60** | Male, 44y, ALL, pre-B-lineage, aplastic marrow. CSF positive for malignant cells. | Induction chemotherapy by Linker et al." and achieved complete remission. Prophylactic IT MTX 12 mg/weekfor6weeks, followed by cranial irradiation at 1800 cGy. Treated with electron beam radiotherapy at 600 cGy An Ommaya reservoir (Heyer-Schulte Corp., Goleta, California) was placed in the cerebrospinal fluid (CSF) space of the frontal convexity, and treatment with IT cytarabine and hydrocortisone. After five IT, patient developed Staphylococcus epidermidis meningitis. Treatment with IT vancomycin and intravenous rifampin was initiated, and IT cytarabine was continued. Completely recovered. | One week after clinical recovery: developed progressive loss of sensorimotor function in the lower extremities and urinary incontinence over a 3- to 4-day period, resulting in complete paralysis below the waist. Ascended over a 2-week period to the fourth cervical dermatome. The patient was quadriplegic but did not require mechanical ventilation. | CSF was negative for ALL involvement and remained negative. MRI normal. Repeated MRI with contrast was performed 3 weeks later: increased T2-weighted and decreased Tl-weighted signals were seen within the spinal cord, with associated expansion from the tip of the conus to the upper cervical spine. Enhancement peripheral cervical and thoracic spinal cord as well as a small focus of enhancement in the midthoracic region. | IT therapy was discontinued (total dose of 580 mg of IT cytarabine, 782mg of IT hydrocortisone, and 12 mg of IT MTX), and there was no evidence of ongoing CNS infection. | Ascending paralysis (myelopathy) into quadriplegia. Remained quadriplegic. He was transferred to a neurorehabilitation unit for ongoing care in died 3 months later. |
| **61** | Female, 70y, lymphoplasmatic lymphoma, bone marrow and kidney. | IT MTX (at a dose of 15 or 12 mg) and cytosine arabinoside (Ara-C, at a dose of 40 or 30 mg) started two months after the first admission. The IT administration was repeated 17 times during the subsequent 8 months, and the total doses of MTX and Ara-C amounted to 243 and 640 mg, respectively. | 7 months after beginning of IT: fever, weakness of the lower extremities and unsteady gait, and many atypical lymphocytes in the peripheral blood, In the next month: flaccid paraplegia and urinary and faecal incontinence. Two months later, the spinal cord lesions progressed to the lower thoracic level, and disorientation also appeared. The consciousness level of the patient gradually deteriorated, and she died the following month, about 14 months after the onset of her illness and about 4 months after the onset of neurological symptoms. | MRI: swelling of the spinal cord below the level of T4. Diffuse low density areas also bilateral cerebral white matter on CT examination. | IT stopped. | Ascending paralysis (myelopathy) 7 months after start IT. Died 4 months after onset of neurological symptoms. **Autopsy:** complete transverse  necrosis (myelomalacia) lower thoracic cord  and vacuolar degeneration of the white matter in the cervical, upper thoracic and lumbo-sacral cord. |
| **Rf** | **Population** | **Initial therapy disease** | **Initial presentation after therapy** | **Diagnostic tools event** | **Treatment after adverse event** | **Conclusion** |
| **62** | Female, 4y, ALL pre-B. | Vincristine, daunorubicin, L-asparaginase, and prednisolone. Weekly IT MTX, cytarabine, and prednisolone (triple IT) during induction chemotherapy. | After three doses triple IT over 3 weeks: incontinence and progressive flaccid paralysis of the legs, weakness of the trunk muscles. Neurological examination revealed areflexia of both legs, with no sensory deficit. | Haematological and serological studies: normal. CSF: 1 white blood cell/μL, absence of malignant cells, normal protein (22 mg/dL) and MBP. NCS consistent with motor lumbosacral polyradiculopathy without peripheral neuropathy; lower extremity velocity and amplitude of compound muscle action potential were normal, F waves were absent. MRI: gadolinium enhancement of the anterior roots at the cauda equina. | Triple IT was stopped, and the patient received I.V. methylprednisolone (30 mg/ kg/day) for 3 days, after which chemotherapy was restarted without triple IT. | Ascending paraplegia (lumbosacral polyradiculopathy). After 4 months of rehabilitation, although the strength of her leg muscles gradually improved, they remained unable to bear weight. |
| **63** | Male, 31y, ALL T cell, complete remission. | Hyper-fractionated cyclophosphamide, vincristine, Adriamycin, and dexamethasone (hyper-CVAD. Relapse: additional hyper-CVAD as well as nelarabine into remission. Patient decided to proceed with a haplo-identical bone marrow transplant. As part of the pre- transplant prophylaxis, he was given systemic hyper-CVAD as well as IT MTX. | One day following the IT infusion of MTX: developed motor weakness and loss of sensation in bilateral lower extremities into complete paraplegia 6days later. Eventually, he developed complete quadriplegia. | CSF: clean. MRI: diffuse increase in signal intensity on T2-weighted imaging extending from T1 to T11. A patchy increase in T2 signal within the right lateral corticospinal tract of the cervical spinal cord extending from C4 to C6. MRI of the brain was unremarkable. | The IT infusion was immediately discontinued, and he was infused with high dose systemic as well as IT steroids. He was treated with high-dose systemic and IT steroids, followed by 5 rounds of plasmapheresis and intravenous immunoglobulins (IVIG), but with no improvement. | Ascending paraplegia into quadriplegia. Subsequently, the patient developed multiple decubitus ulcers leading to refractory septic shock 5 months later. |
| **64** | **1pt:** Male, 17y, ALL, **2pt:** Male, 40y, Hodgkin lymphoma. | **1pt**: Autologous peripheral blood stem cell transplantation (PBSCT). IT MTX 12.5 mg. After confirming CNS relapse, he was given IT MTX 12.5 mg, Ara-C 40 mg and dexamethasone 4 mg about one week after the first IT MTX. **2pt:** Treated with Berlin-Frankfurt-Munster (BFM) protocol, I.V. MTX, and IT MTX and Ara-C. Autologous (PBSCT). | **1pt:** Soon after completion of IT injection (45 min): numbness of his left lower limb, development of paraplegia and paraesthesia of the lower parts of the body. No urinary or bladder incontinence. BP was high (200/120 mmHg) and tachycardic, pulse rate of 120 beats per minute. Sensory loss was noted from lower extremities up to T4. **2pt:** Sudden onset of paraplegia and urinary retention two days after the fifth IT MTX during the second BFM cycle A. Four days after the event: Glasgow Coma Scale (GCS) from 15/15 to 10/15 and developed respiratory distress. He was electively intubated for respiratory distress and poor GCS. | **1pt**: contrast-enhanced CT of the brain was done on the same day and was normal. Repeated CSF examination during the neurological event was unremarkable. **2pt:** Repeat MR imaging: no disease progression, spinal cord compression or brain metastasis. He continued to deteriorate and succumbed ten days later. CSF: unremarkable. Contrast CT brain on the same day: unremarkable. | **1pt:** The symptoms completely resolved 30 minutes after IV hydrocortisone 100 mg and piriton 10 mg. **2pt:** N.A. | **1pt:** Complete paraplegia within 1 week after IT. Resolved in 30 min day after I.V. hydrocortisone. **2pt:** paraplegia and passed away in 10 days. |
| **Rf** | **Population** | **Initial therapy disease** | **Initial presentation after therapy** | **Diagnostic tools event** | **Treatment after adverse event** | **Conclusion** |
| **65** | Male, 8y, ALL, meningeal relapse. | Prophylaxis IT MTX and irradiation of the CNS (20 Gy. Treatment was limited to IT chemotherapy administered on a biweekly basis (MTX, aracytine, prednisone). | Transverse myelitis occurred three days after the eighth injection and resulted in permanent paraplegia. | CSF MBP monitoring the whole IT treatment. His CSF MBP levels had always remained low, and below 2 ng/mL. | N.A. | Permanent paraplegia. Their conclusion: Thus, CSF MBP monitoring apparently does not provide protection against such accidents. |
| **66** | Female 12y, ALL (non-B non-T). Remission for 3 years. | Treatment according to the “SAKK 1976, low risk” protocol, including CNS prophylaxis and 1 course of 1800 R CNS irradiation and 6 IT injections of MTX (50 mg total dose). Cumulatively, the patient received 1.7 g MTX oral, 6.7 g MTX I.V, and 0.35 g MTX IT in 26 IT. injections. | The first signs 10 hrs after TIT: pain in both legs and developed a high- grade fever (39°C). Over 36hr symptoms progressed to a severe meningeal syndrome with violent headaches, opisthotonos, and walking difficulties. Within 48 hr definite paraparesis, there was no Babinski sign and no sphincter disturbances. Complete flaccid paraplegia within 4 days. Preservation of deep and superficial sensation. On the 5^th^ day went into a coma (GS 3). | Myelography and (CT) normal. A complete search for viral, bacterial, or fungal infections in the blood, CSF, stool, and urine was negative: Blood and CSF serology and cultures for Toxoplasma gondii, influenza B, mumps, Epstein-Barr virus (EBV), cytomegalovirus (CMV), and adenovirus remained negative, and no evidence of CNS relapse could be found. A measurement of MTX level in the CSF was made on day 8 after TIT; the drug was below any detectable level. | N.A. | Paraplegia into coma within 9 days. Passed away by stopping the pulmonary ventilator. Autopsy: no signs of increased intracranial pressure. Thoracolumbar spinal transverse necrosis. Widespread subpial and subependymal micro vacuolization, necrosis. |
| **Rf** | **Population** | **Initial therapy disease** | **Initial presentation after therapy** | **Diagnostic tools event** | **Treatment after adverse event** | **Conclusion** |
| **67** | Female, 41y, ALL  treatment of CNSL after allogeneic hematopoietic stem cell transplantation. | 4^th^ IT MTX 15 mg, Ara-C 50 mg, and dexamethasone 5 mg. Normal saline 2.0 ml was used as the dilution for MTX and Ara-C, respectively. Lidocaine (2%) 1.5 ml was used for local infiltration. | Shortly after IT: developed complete sensorimotor paraplegia in the lower extremities, which ascended and affected both upper limbs gradually.  Physical examination showed that the block level subsided to T4, T8, and then T10 about 60, 100, and 150 min after IT. Areflexia and flaccid without any sensation below these block levels. The patient got agitated because of paroxysmal pruritus over the area from both upper extremities to umbilical level (T10). | Creatine kinase level of 6493 U/L, with patient.  A repeat blood creatine  kinase showed 3162 U/L.  MTX dose in the drained CSF was estimated by colorimetry at a wavelength of 294 nm and 4.1 mg MTX was retrieved. | The patient was placed in reclining position and oxygen delivered as she felt dyspnoea. Promethazine 25 mg and dexamethasone 5.0 mg were intravenously injected with little effect. CSF replacement. Diazepam 10 mg was intravenously injected (for general muscles jerks during CSF replacement). | Acute paraplegia into quadriplegia. Three days after the CSF replacement,  the patient could walk freely in the ward. A 3-month follow-up revealed that she recovered uneventful. |
| **68** | **1pt:** M, 12y, Burkitt  **2pt:** M, 9y, Burkitt  **3pt:** M, 16y, Burkitt  **4pt:** F, 16y, Burkitt  **5pt:** M, 9y, Burkitt  **6pt:** M, 11y, Burkitt  **7pt:** F, 16y, AML/ALL biphenotypic  **8pt:** F, 12y, CNS lymphoma  **9pt**: F, 4y, Burkitt | **1pt:** IT MTX, Ara-C, hydrocortisone and HD MTX, Thiotepa.  **2pt:** IT MTX, Ara-C and hydrocortisone and CRT.  **3pt:** IT MTX, Ara-C.  **4pt:** IT MTX, Ara-C and hydrocortisone and CRT.  **5pt:** IT MTX, Ara-C and hydrocortisone and CRT.  **6pt:** IT MTX, Ara-C and hydrocortisone.  **7pt:** IT MTX, Ara-C.  **8pt:** IT MTX, Ara-C and spinal radiation.  **9pt**: IT MTX, Ara-C and spinal radiation. | All presented with ascending paraplegia. Symptoms specified below:  **1pt:** Onset: 7d, cord destroyed below T6.  **2pt:** Onset: 29d, ascending to brain.  **3pt:** Onset: 42d, paraparesis.  **4pt:** Onset: 42d, paraplegia.  **5pt:** Onset: 8w, paraplegia.  **6pt:** Onset: 11w, paraparesis.  **7pt:** Onset: 11w, quadriplegia.  **8pt:** Onset: 12w, paraplegia.  **9pt**: Onset: 13w, paraplegia. | **1pt:** CSF: negative.  **2pt:** CSF: positive.  **3pt:** CSF: negative.  **4pt:** CSF: negative.  **5pt:** CSF: positive.  **6pt:** CSF: positive.  **7pt:** CSF: negative.  **8pt:** CSF: negative.  **9pt** CSF: negative. | N.A. | **1pt:** Partial recovery.  **2pt:** Death due tox.  **3pt:** Partial recovery.  **4pt:** Partial recovery.  **5pt:** Slowly recovering.  **6pt:** Partial recovery.  **7pt:** Partial recovery (DOD).  **8pt:** Partial recovery (DOD).  **9pt**: recovery. |
| **Rf** | **Population** | **Initial therapy disease** | **Initial presentation after therapy** | **Diagnostic tools event** | **Treatment after adverse event** | **Conclusion** |
| **69** | Female, 46y, ALL | Prednisone, 6 MP, cytosine-arabinoside and later vincristine. Five IT injections of 20 mg MTX with parabens preservative in 20 ml of normal saline were administered over a 2-week period. | 1d after the last IT MTX: weakness and pains in both legs. Flaccid paraplegia with a sensory level at D10 within 3 weeks, stool and urinary incontinence, right arm weakness. | N.A. | N.A. | Died of sepsis. |
| **70** | Male, 15y, Burkitt. | 1 cycle of cyclophosphamide, vincristine, and prednisolone;  2 cycles of cyclophosphamide, vincristine, prednisolone, doxorubicin, and MTX; and 1 cycle of etoposide with HD cytarabine. Additional: 2 sessions of HD systemic MTX of 14 g. 70 mg of IT cytarabine and 35 mg of hydrocortisone followed by a total of 8 IT triple therapy of 15 mg of MTX, 30 mg of cytarabine, and 15 mg of hydrocortisone. Due to CNS relapse: additional triple IT. | Between the 10th and 11th round IT”:  difficulty urinating, developed gait ataxia and bilateral lower extremity weakness. Proprioception and sense of light touch were decreased in the  bilateral lower extremities. Cerebellar function tests normal. Nine days after the symptom onset, the 11th IT triple therapy was administered. Day 27: bilateral facial palsy and decreased facial sensation developed. Progressed to upper extremities. | MRI: diffuse high signal intensities along the  dorsal columns of the whole spinal cord on T2 images indicating subacute combined degeneration. Serum  B12 normal (3354 ng/L) and serum copper was decreased (524 mg/L). NCS facial neuropathy. 61st day after onset showed increased extent of the high signal intensities to the bilateral lateral columns of the thoracic spinal cord on T2-weighted imaging. | Copper (II) sulphate pentahydrate 4 mL was given over 15 minutes I.V based on the suspicion that reduced copper level might have induced his neurologic condition. | Ascending paraplegia due to myelopathy. Eventually died of respiratory failure. |
|  | **Re-run** |  |  |  |  |  |
| **Rf** | **Population** | **Initial therapy disease** | **Initial presentation after therapy** | **Diagnostic tools event** | **Treatment after adverse event** | **Conclusion** |
| **71** | Male, 28y, T-ALL, CNS involvement. | Hyper-CVAD, 12 doses of alternating IT MTX and cytarabine. 20 sessions of radiotherapy to mediastinum. Maintained on POMP (6-mercaptopurine, vincristine, MTX and prednisone). **For recurrence**: BFM protocol (vincristine, prednisone, l-asparaginase, daunorubicin) + triple IT chemotherapy (MTX 12 mg, cytarabine 100 mg and dexamethasone 8 mg). | Day 15 BMF: tingling sensation and weakness of lower extremities bilaterally, ascended over three days to hips. Decreased motor power (3/5), absent deep tendon reflexes and absent Babinski bilaterally. The rest of neurological exam, including the sensory exam, was normal. Developed urinary retention as well as sensory deficit of light-touch, pinprick and vibration sensation up to the T10 dermatome. | MRI no changes, no CSF at this point. **Later:** CSF revealed no cells, normal glucose (68 mg/dL), elevated protein (77 mg/dL) and borderline LDH (39 U/L). Infectious work-up of the CSF including gram and fungal stains, cultures, TB PCR as well as meningitis PCR panel (including HSV-1, HSV-2, VZV, EBV, CMV, HHV-6, Enterovirus, Cryptococcus) all negative. EMG was suggestive of central pathology. The patient also had low serum folate (1.7 pg/mL) and normal vitamin B12 (704 pmol/L) levels.  **31 days later:** MRI of the spine was repeated: Sagittal STIR image of cervical and thoracic spine showing high T2 signal from C1 to T8, Axial T2W image showing expansion of the spinal cord with an abnormal signal involving most of the cross-sectional area of the cervical cord. | Gabapentin 300 mg three times per day. Folate replenishment. IV methylprednisolone 1 mg/Kg daily. 10 mg/Kg along with IV leucovorin 20 mg four times per day and intramuscular cyanocobalamin 100mcg daily. | Myelopathy and no Improvement. |
| **72** | Girl, 15y, T-ALL. | Cyclophosphamide, vincristine, doxorubicin, and dexamethasone. IT MTX and Ara-C injections were also given for central nervous system (CNS) prophylaxis. | 3 days after 6th IT: sudden onset of bilateral lower limbs weakness. Neurological examinations revealed flaccid paraparesis with muscle power of ⅗. 2 weeks later: worsening bilateral lower limbs' weakness and urinary retention. Muscle power of the lower limbs was ⅖ with bilateral extensor plantar reflexes. | Onconeural antibodies not detected. NCS performed 5 days later showed a length-dependent sensorimotor axonal neuropathy secondary to vincristine use. CT brain and MRI of the lumbosacral spine were normal. (PET)-CT of whole body showed complete metabolic response with tumor regression. The repeated spine MRI revealed inflammatory changes in the lower thoracic and upper lumbar region. | IVIG for GBS. A 5-day course of IV methylprednisolone was given, followed by a short course of oral prednisolone. | 2 weeks after IVIG, progressed. Her condition gradually improved with rehabilitation. At 6-months follow up, she was able to ambulate with assistance and her lower limbs power improved to ⅘. |
| **73** | Female, 31y, AML, CNS involvement. | Induction idarubicin and cytarabine (I3A7); bone marrow and CSF positive -> IT MTX, total accumulative dose during the following 10 months since the initial diagnosis was 120 mg (12 mg × 10 sessions);  haploidentical bone marrow transplantation, followed by haploidentical peripheral blood stem cell transplantation (complete remission) | 6 weeks after the last IT MTX:  bilateral lower limb symmetric numbness without shooting pain. Increasing weakness of the lower extremities developed from the distal and gradually progressed upwards; urinary incontinence and severe low back pain. Decreased pain and proprioception below L4 level and absence below S3. Deep tendon reflexes were hyperactive over bilateral lower extremities, accompanied with bilateral ankle clonus. Muscle power of bilateral proximal and distal legs was 2 and 1 on a scale of 1 to 5, respectively. | MRI: bilateral symmetrical signal change in spinal cord, predominately in the posterior and lateral columns. | Leucovorin, prednisone and vitamins B12 were administered for rescue treatment. Physical therapy was also arranged. | Before discharge, she was able to walk over 100 m without device assistance. |
| **74** | Male, 23y, ALL. | LAL1913 protocol; On protocol day 1, he also underwent CNS prophylaxis with one IT administration of 12.5 mg MTX and dexamethasone. On day 13, the whole regimen protocol was discontinued for the onset of neutropenia and thrombocytopenia associated to fever and necrotizing fasciitis in the left shoulder. The patient was treated with antibiotic therapy and surgery. | 23 days after first IT: complained with lower limb weakness. Neurological examination showed flaccid paraplegia: hip flexion (MRC 1/5 on the left, 2/5 on the right). Deep tendon reflexes were absent. No sensory, sphincter, and/or autonomic disturbances were detected. MRC sum score for lower limbs was 3/30. | CSF after 25 days after symptom onset: protein values (170 mg/dL) and normal cell count (< 5/μl). Extensive laboratory screening, including virological tests and antiganglioside antibodies, resulted normal. (MRI) of the spinal cord revealed thickening and increased T2 signal with gadolinium enhancement in the cauda equina ventral roots. EMG showed abnormalities in the left tibialis anterior and vastus lateralis, with paraspinal sparing. | IVIG 0.4 g/kg/day for 5 consecutive days, followed by high-dose IV methylprednisolone 1 g/day for 5 consecutive days and intensive physical rehabilitation. | Three months after symptom onset, the patient was able to walk with bilateral support, with steppage. |
| **75** | Sex undefined, 72y, B-ALL. CNS involvement. | Imatinib and dasatinib; 6 cycles of vincristine, cyclophosphamide, etoposide and velcade; and 12 monthly IT (IT) MTX (MTX) injections of 12.5 mg with folic acid rescue. -> asymptomatic remission for 1 year  2)  After a secondary leptomeningeal involvement by leukaemia was confirmed: he received 50 mg IT cytosine arabinose (Ara-C) with dexamethasone PO 8 mg for 3 days without immediate complications | 2) In 2 days, he developed severe painless flaccid paraplegia, leg areflexia, bilateral upgoing plantar response, spinal sensory deficit to T10 level, and partial urinary incontinence. | MRI showed degenerative changes and a subtle intramedullary no enhancing T2-signal prolongation from D11 to conus medullaris.  CSF yielded elevated protein, malignant lymphocytes, and positive BCR-ABL and B cell immunoglobulin gene rearrangement, confirming the diagnosis of a secondary leptomeningeal involvement by leukemia.  Second MRI demonstrated worsening of the no enhancing intramedullary T2-prolongation with cord expansion extending from D8-D9 to conus medullaris. CSF analysis showed normal protein and glucose, 50 polymorphonuclear cells, and negative cytology, culture and negative PCR for HSV1, HSV2, EBV, CMV, enterovirus, VZV, JC-virus and HHV6. CSF oligoclonal bands were positive, and paraneoplastic panel with anti-Hu, anti-Yo, and anti-Ri was negative. | Because of the subacute onset, myelitis was clinically suspected, and treatment with IV solumedrol was initiated. Third MRI showed partial resolution if MRI findings. Ommaya reservoir was inserted for further administration of IT MTX, without Ara-C. | The patient died from massive pulmonary emboli with cardiorespiratory failure. After a week of steroids, he regained antigravity muscle strength in the legs, but could not stand. |
| **76** | Male, 21y, B-ALL, expressing CD19, CD10 and CD81. CNS involvement. | He was treated with cyclophosphamide, daunorubicin, vincristine and prednisone (CALGB regimen) for 6 months. Three months before admission, he had started early intensification with IT MTX, IV cyclophosphamide, mercaptopurine, cytarabine, vincristine and L-asparaginase because of the high recurrence risk related to a leucocytosis over 30 000 cells/mm3. MADIT IT regimen (MTX, arabinoside and dexamethasone) and fludarabine, idarubicin, granulocyte colony-stimulating factor and cytarabine systemic chemotherapy. | His neurological symptoms began 20 days after starting IT MADIT. Bilateral numbness in the legs with gait impairment, followed by urinary incontinence. Sensory abnormalities in both legs to the umbilicus. Mild distal weakness of the legs; deep tendon reflexes were normal in the upper limbs and at the knee, but both ankle reflexes were absent and plantar responses extensor. There was markedly impaired joint position and vibration sense in both legs, with impaired pain and temperature sensation below the sixth thoracic dermatome. His gait was ataxic, resembling a tabetic gait and Romberg sign was present. | MR scan of the spinal cord showed bilateral symmetric, and extensive T2/FLAIR increased signal involving the dorsal columns in the thoracic cord, with no contrast enhancement, restricted diffusion or mass effect Serum vitamin B12, copper and zinc concentrations were normal. Serum folate was at the lower end of the normal range at 3.5 µg/L (3.0–17.0). CSF examination was normal. | Interrupted IT chemotherapy and gave folate. After a few days, there was progressive improvement in proprioception, gait and distal leg weakness. We gave inotuzumab, IT cytarabine and dexamethasone, and prepared for bone marrow transplantation. | Two months later, he could walk unassisted.  Six months later, a follow-up bone marrow biopsy identified 2% blast cells and CSF showed 52% blast cells, despite there being no neurological symptoms. However, his condition progressed, with massive brain oedema with CNS infiltration and death. |
| **77** | Boy, 14y, T-ALL. | He achieved complete remission with induction chemotherapy. Subsequently, he received early intensification therapy followed by NEL (650 mg/m2/day for 5 days). Five weeks after treatment with NEL, he was given high-dose MTX followed by IT MTX 1 week later. | One week after IT MTX, he developed somnolence, drooling, dysarthria, and left arm paralysis. The symptoms disappeared spontaneously in 3 days. One week after disappearance of the symptoms, the patient showed loss of sensation in the left leg and weak left ankle dorsiflexion. In the standing position, the patient lost balance with eye closure, and thus Romberg's sign was present. The patient stood with a wide stance and could not walk without looking down at his feet. | Diffusion-weighted imaging (DWI) showed high-intensity regions with a reduced apparent diffusion coefficient in the centrum semiovale, bilaterally Brain MRI 1 week after IT MTX administration shows high-intensity regions He was diagnosed with MTX leukoencephalopathy.  **Re-appearance of symptoms:**  On re-examination, MRI showed a subtle high intensity region in the right centrum semiovale, and the dimensions and diffusion changes had regressed from those at the onset of the neurological symptoms. Deep sensation was gradually lost in both legs, with preservation of superficial sensation.  Spinal MRI showed a longitudinal, high-intensity region from Th4 to the cone in the dorsal column on T2-weighted images on the 47th day after onset of loss of leg sensation shows a longitudinal high-intensity region from Th4 to the cone in the dorsal column | Started on oral folate and vitamin B12. Examination of the serum sample stored before folate and vitamin B12 was started showed normal levels of folate, vitamin B12, and homocysteine. | The sensory loss and gait disturbance gradually improved and neurological symptoms resolved 6 months after onset. The high intensity in the dorsal column gradually diminished and disappeared 1.5 years after onset. The patient showed complete recovery of neurological symptoms and magnetic resonance imaging (MRI) abnormalities. and his leukemia was in remission |

1: Alsdorf, W, 2: Amer-Salas, N, 3: Anderson, S, 4: Ateşoǧlu, E, 5: Aytac, S, 6: Badke, C, 7: Bellon, J, 8: Benesch, M, 9: Breuer, A, 10: Byrnes, D, 11: Cachia, D, 12: Castillo-Torres, S, 13: Chukwu, B, 14: Cyriac, S, 15: Dara, A, 16; Dornbos, D, 17; Dufourg, M, 18: Dunton, S, 19: Gagliano, R, 20: Gallego, P, 21: Garcia-T, J, 22: Geissler, R, 23: Gosavi, 24: Grisold, W, 25 Han, A, 26: Hilgendorf, I, 27: Jabbour, E, 28 Jordan, B, 29: Joseph, P, 30: Jurczak, W, 31: Keidan, I, 32: Kleinschmidt-DeMasters, B, 33: Koh, S, 34 Lakhani, A, 35: Lalayanni, C, 36: Lapucci, C, 37: Lee, E, 38: Lee, H, 39: Lewis, K, 40: Lu, C, 41: Maramatton, B, 42: McLean, D, 43; Mena, H, 44: Montejo, C, 45: Murata, K, 46: Ngo, D, 47: Ostermann, K, 48: Özön, A, 49: Park, S, 50: Pascual, A, 51: Pinnix, C, 52: Pisani, F, 53: Resar, L, 54: Rizzieri, D, 55: Rolf, N 56: Saito, F, 57: Saleh, M, 58: Santos-Garcia, D, 59: Schwenn, M, 60: Sherman, P, 61: Shintaku, M, 62: Suematsu, M, 63: Tariq, H, 64: Teh, H, 65: Thyss, A. 66: Von der Weid, N, 67: Wang, Z, 68: Watterson, J, 69: Weiss, S, 70: Yi, Y **Re-run:** 71: Bidikian, A.H, 72: Cheong, X. K, 73: Chiu, Y.H, 74: Landolfi, A. 75: Mikhelashvili, L, 76: Rodriquez, P.G.B, 77: Sawamura, F.

Articles in red represent similar cases but were excluded in the final data analysis because of missing data.

**Table S4:** Diagnostic overview as indication of PIPS compared to other diagnoses.

|  | | |
| --- | --- | --- |
| **Diagnostics** | **Implication** | **PIPS** |
| **Serum/Blood test** | | |
| **Blood, leucocytosis, CRP, ESR** | **Infection/Inflammation** | **Non-specific** |
| Antibodies: HIV, mycoplasma, arbovirus, Bartonella Henselae, Lyme’s disease, Blood culture: bacterial, viral | Infectious- postinfectious syndromes | Negative |
| ANA, anti-dsDNA, ENA, ds-DNA, ANCA, antiphospholipid antibody, LAC | Systemic autoimmunity | Negative |
| Anti-AQp4 | Neuromyelitis Optica | Negative |
| Anti-MOG | MOG-associated disease | Negative |
| ACE-level | Sarcoidosis | Negative |
| Vitamin B12, folic acid, E, biotidinase, copper, ammonium, amino acids, lactate | Deficiencies, metabolic diseases associated with paralysis | Normal (~95%) |
| **CSF analysis** | | |
| Cytology, differentiation, protein, glucose levels | Infection, inflammation, malignancy | Negative for cells,  Protein normal in ->50%, occasionally increasing after weeks.  Glucose normal |
| Microorganisms, PCR | Infection: Entero-, parecho-, herpes simplex-, Epstein-Barr-, varicella zoster-, influenza-, hepatitis A, B en C-virus Human T-lymphotropic virus type 1, human herpesvirus type 6 en 7, Mycoplasma pneumonia, Bartonella Henselae, Borrelia burgdorferi, mycoplasma, tuberculosis | Negative |
| IgG-index, oligoclonal bands (related to serum) | Deviating in multiple sclerosis, neuromyelitis optica, and autoimmune myelitis | Negative |
| **Other** | | |
| Virus PCR e.g., respiratory or enterovirus in feces | Infectious or para-infectious syndromes | negative |
| **MRI** |  |  |
| MRI: cranial, spine, and peripheral nerves including T2. | Compression, signs of inflammation, neurodegenerative diseases | Non-specific, negative (🡪 60% cases) at time of onset of PIPS. |
| ACE=angiotensin converting enzyme, ANA=antinuclear antibody, ANCA=antineutrophil cytoplasmatic antibody, anti-dsDNA=anti-double stranded-DNA, AQP4=aquaporine-4, ESR=erythrocyte sediment rate, CRP=C-reactive protein,  ENA=extractable nuclear antigen, HIV=human immunodeficiency virus, PIPS = Post intrathecal paraplegia syndrome, LAC=lupus anticoagulants, MOG=myeline oligodendrocyte glycoprotein. | | |

**References**

64. Anderson SC, Baquis GD, Jackson A, Monteleone P, Kirkwood JR. Ventral polyradiculopathy with pediatric acute lymphocytic leukemia. *Muscle Nerve*. 2002;25(1):106-110. doi:10.1002/mus.1219

65. Birtaş Ateşoğlu E, Fıratlı Tuğlular T, Adıgüzel C, et al. A case of Cauda Equina syndrome in a leukemic patient due to intrathecal methotrexate. *Turk J Haematol*. 2007;24(1):28-31. http://www.ncbi.nlm.nih.gov/pubmed/27263482

66. Bellon JR, Smith AS, Cohen ML. Ascending cord necrosis. Complication of intrathecal chemotherapy with radiologic-pathologic correlation. *Clin Pediatr (Phila)*. 1995;34(9):506-509. doi:10.1177/000992289503400911

67. Breuer AC, Pitman SW, Dawson DM, Schoene WC. Paraparesis following intrathecal cytosine arabinoside: a case report with neuropathologic findings. *Cancer*. 1977;40(6):2817-2822. doi:10.1002/1097-0142(197712)40:6<2817::aid-cncr2820400611>3.0.co;2-v

68. Byrnes DM, Vargas F, Dermarkarian C, et al. Complications of Intrathecal Chemotherapy in Adults: Single-Institution Experience in 109 Consecutive Patients. *J Oncol*. 2019;2019:4047617. doi:10.1155/2019/4047617

69. Cyriac S, Rajendranath R, Sagar TG. Early CNS toxicity after intrathecal methotrexate. *Indian J Hematol Blood Transfus*. 2008;24(4):186-187. doi:10.1007/s12288-008-0045-4

70. Gagliano RG, Costanzi JJ. Paraplegia following intrathecal methotrexate: report of a case and review of the literature. *Cancer*. 1976;37(4):1663-1668. doi:10.1002/1097-0142(197604)37:4<1663::aid-cncr2820370408>3.0.co;2-7

71. Gállego Pérez-Larraya J, Palma JA, Carmona-Iragui M, et al. Neurologic complications of intrathecal liposomal cytarabine administered prophylactically to patients with non-Hodgkin lymphoma. *J Neurooncol*. 2011;103(3):603-609. doi:10.1007/s11060-010-0428-x

72. García-Tena J, López-Andreu JA, Ferrís J, et al. Intrathecal chemotherapy-related myeloencephalopathy in a young child with acute lymphoblastic leukemia. *Pediatr Hematol Oncol*. 1995;12(4):377-385. doi:10.3109/08880019509029588

73. Geissler RG, Bergmann L, Hacker H, Knupp B, Walker B, Hoelzer D. Severe central nervous adverse effect of intrathecal chemotherapy in a 16-yr-old patient with Burkitt’s type lymphoma. *Eur J Haematol*. 1997;58(4):286-288. doi:10.1111/j.1600-0609.1997.tb01668.x

74. Lakhani AK, Zuiable AG, Pollard CM, Milne A, Treleaven J, Powles RL. Paraplegia after intrathecal mitozantrone. *Lancet*. 1986;2(8520):1393. doi:10.1016/s0140-6736(86)92030-1

75. Lee HY, Im S il, Kang MH, et al. Irreversible paraplegia following one time prophylactic intrathecal chemotherapy in an adult patient with acute lymphoblastic leukemia. *Yonsei Med J*. 2008;49(1):151-154. doi:10.3349/ymj.2008.49.1.151

76. Montejo C, Navarro-Otano J, Mayà-Casalprim G, Campolo M, Casanova-Mollá J. Acute lumbar polyradiculoneuropathy as early sign of methotrexate intrathecal neurotoxicity: Case report and literature review. *Clin Case Rep*. 2019;7(4):638-643. doi:10.1002/ccr3.2026

77. Ozön A, Topaloğlu H, Cila A, Günay M, Cetin M. Acute ascending myelitis and encephalopathy after intrathecal cytosine arabinoside and methotrexate in an adolescent boy with acute lymphoblastic leukemia. *Brain Dev*. 1994;16(3):246-248. doi:10.1016/0387-7604(94)90079-5

78. Park S, Kang JI, Bang H, Kim BR, Lee J. A case of the cauda equina syndrome associated with the intrathecal chemotherapy in a patient with primary central nervous system lymphoma. *Ann Rehabil Med*. 2013;37(3):420-425. doi:10.5535/arm.2013.37.3.420

79. Pascual AM, Coret F, Casanova B, Láinez MJA. Anterior lumbosacral polyradiculopathy after intrathecal administration of methotrexate. *J Neurol Sci*. 2008;267(1-2):158-161. doi:10.1016/j.jns.2007.09.035

80. Pisani F, Scarano A. Acute motor neuropathy after intrathecal treatment with methotrexate: A case report. *Journal of Pediatric Neurology*. 2015;05(03):247-249. doi:10.1055/s-0035-1557383

81. Resar LM, Phillips PC, Kastan MB, Leventhal BG, Bowman PW, Civin CI. Acute neurotoxicity after intrathecal cytosine arabinoside in two adolescents with acute lymphoblastic leukemia of B-cell type. *Cancer*. 1993;71(1):117-123. doi:10.1002/1097-0142(19930101)71:1<117::aid-cncr2820710119>3.0.co;2-k

82. Rolf N, Boehm H, Kaindl AM, Lauterbach I, Suttorp M. Acute ascending motoric paraplegia following intrathecal chemotherapy for treatment of acute lymphoblastic leukemia in children: case reports and review of the literature. *Klin Padiatr*. 2006;218(6):350-354. doi:10.1055/s-2006-942276

83. Schwenn MR, Blattner SR, Lynch E, Weinstein HJ. HiC-COM: a 2-month intensive chemotherapy regimen for children with stage III and IV Burkitt’s lymphoma and B-cell acute lymphoblastic leukemia. *J Clin Oncol*. 1991;9(1):133-138. doi:10.1200/JCO.1991.9.1.133

84. Suematsu M, Imamura T, Chiyonobu T, Osone S, Hosoi H. Lumbosacral polyradiculopathy after intrathecal chemotherapy in pediatric acute lymphoblastic leukemia. *Int J Hematol*. 2018;107(5):499-501. doi:10.1007/s12185-018-2427-1

85. Thyss A. Myelin basic protein in CSF of children receiving intrathecal chemotherapy. *J Clin Oncol*. 1986;4(10):1569. doi:10.1200/JCO.1986.4.10.1569

86. Watterson J, Toogood I, Nieder M, et al. Excessive spinal cord toxicity from intensive central nervous system-directed therapies. *Cancer*. 1994;74(11):3034-3041. doi:10.1002/1097-0142(19941201)74:11<3034::aid-cncr2820741122>3.0.co;2-o

87. Cheong XK, Ng CF, Tan HJ, Remli R. Acute Thoracolumbar Myelitis Secondary To Intrathecal Chemotherapy. *J Neurosci Rural Pract*. 2021;12(4):815-816. doi:10.1055/s-0041-1735246

88. Landolfi A, Vinciguerra C, Diana F, et al. Anterior lumbosacral polyradiculoneuropathy following intrathecal methotrexate administration: a case report and literature update. *Neurol Sci*. 2023;44(2):715-718. doi:10.1007/s10072-022-06520-y
